# Functions of Oxysterol-Binding Proteins at Membrane Contact Sites and Their Control by Phosphoinositide Metabolism

**DOI:** 10.3389/fcell.2021.664788

**Published:** 2021-06-24

**Authors:** Fubito Nakatsu, Asami Kawasaki

**Affiliations:** Department of Neurochemistry and Molecular Cell Biology, Niigata University School of Medicine and Graduate School of Medical/Dental Sciences, Niigata, Japan

**Keywords:** ORPs, phosphoinositide, membrane contact site (MCS), lipid transfer protein (LTP), PI4P, phosphatidylserine (PS), cholesterol, lipid countertransport

## Abstract

Lipids must be correctly transported within the cell to the right place at the right time in order to be fully functional. Non-vesicular lipid transport is mediated by so-called lipid transfer proteins (LTPs), which contain a hydrophobic cavity that sequesters lipid molecules. Oxysterol-binding protein (OSBP)-related proteins (ORPs) are a family of LTPs known to harbor lipid ligands, such as cholesterol and phospholipids. ORPs act as a sensor or transporter of those lipid ligands at membrane contact sites (MCSs) where two different cellular membranes are closely apposed. In particular, a characteristic functional property of ORPs is their role as a lipid exchanger. ORPs mediate counter-directional transport of two different lipid ligands at MCSs. Several, but not all, ORPs transport their lipid ligand from the endoplasmic reticulum (ER) in exchange for phosphatidylinositol 4-phosphate (PI4P), the other ligand, on apposed membranes. This ORP-mediated lipid “countertransport” is driven by the concentration gradient of PI4P between membranes, which is generated by its kinases and phosphatases. In this review, we will discuss how ORP function is tightly coupled to metabolism of phosphoinositides such as PI4P. Recent progress on the role of ORP-mediated lipid transport/countertransport at multiple MCSs in cellular functions will be also discussed.

## Introduction

Lipids have multiple essential roles, including serving as building blocks for cellular membranes, storing energy, and regulating signaling and membrane dynamics/trafficking. In eukaryotes, most, but not all, lipids are synthesized at the endoplasmic reticulum (ER) and then must be correctly delivered to the places where they exert diverse functions (van Meer and de Kroon, 2011; Santos and Preta, 2018). In addition, lipids often move and change their location even during their metabolic or catabolic processes (van Meer et al., 2008). Thus, lipids rely on their transport systems for accomplishing their diverse and complex tasks in biological systems.

Lipids are transported within cells via membrane carriers (vesicles) along secretory and endocytic membrane trafficking pathways (van Meer et al., 2008; Vance, 2014; Stefan et al., 2017). In addition to vesicular transport, lipids are also transported in a vesicle-independent manner (Holthuis and Levine, 2005; Lev, 2012; Reinisch and Prinz, 2021). Non-vesicular lipid transport is mediated by so-called lipid transfer proteins (LTPs) (Holthuis and Menon, 2014; Wong et al., 2019). LTPs contain a hydrophobic cavity that sequesters lipid molecules from aqueous cytosolic environments, and in this way, they mediate lipid transport between cellular membranes (Wong et al., 2017). Although LTPs are technically able to transport lipids to any accessible place by freely moving in the cytosol, they often do so at membrane contact sites (MCSs). MCSs are places where there is close apposition of cellular membranes (generally 10–30 nm, but the distance differs depending on the type of MCSs) (Helle et al., 2013; Eisenberg-Bord et al., 2016; Scorrano et al., 2019). Accumulating evidence indicates that the ER, which is widely distributed throughout the cell, forms MCSs with most of the organelles or the plasma membrane (PM), and these MCSs serve as zones for non-vesicular lipid transport (Phillips and Voeltz, 2015; Wu et al., 2018; Balla et al., 2019; Prinz et al., 2019; Bohnert, 2020). A number of LTPs have been reported to localize at MCSs and, thus, mediate the transport of a variety of lipid ligands (Wong et al., 2017, 2019).

In this review, we provide an overview of recent progress on understanding the role of oxysterol-binding protein (OSBP)-related proteins (ORPs) (Raychaudhuri and Prinz, 2010; Olkkonen, 2015; Pietrangelo and Ridgway, 2018), a large family of LTPs. Particular focus is placed on their lipid transport function at MCSs and their role in cellular processes in mammals. Given that ORP functions are closely coupled to phosphatidylinositol 4-phosphate (PI4P) metabolism, we will begin with background information as well as recent findings on PI4P, followed by ORP family functions, with the overall goal of an in-depth discussion on the physiological significance of lipid transport at MCSs mediated by ORPs and phosphoinositides.

## Phosphatidylinositol 4-Phosphate

### Phosphatidylinositol 4-Phosphate Metabolism in the Cell

Phosphoinositides are a minor group of phospholipids that represent 10–15% of total phospholipids in the cell (Vance, 2014). The inositol ring in their headgroup is exposed to the cytosol, and its 3, 4, or 5 position can be phosphorylated or dephosphorylated to create seven distinct phosphoinositide species (Balla, 2013). Those phosphoinositides are unevenly distributed in the cell (Di Paolo and De Camilli, 2006). For instance, PI(4,5)P_2_ is concentrated at the PM, while PI4P, its major precursor, is distributed more widely (see below for details). PI(3,4,5)P_3_ is also localized at the PM, but its level transiently increases locally under certain conditions. Such spatial and temporal distribution of each phosphoinositide species, which determines the identity of cellular membranes, is tightly controlled, mostly based on the action of their metabolic phosphoinositide kinases or phosphatases that localize in distinct cellular compartments (Di Paolo and De Camilli, 2006; Balla, 2013).

**FIGURE 1:**
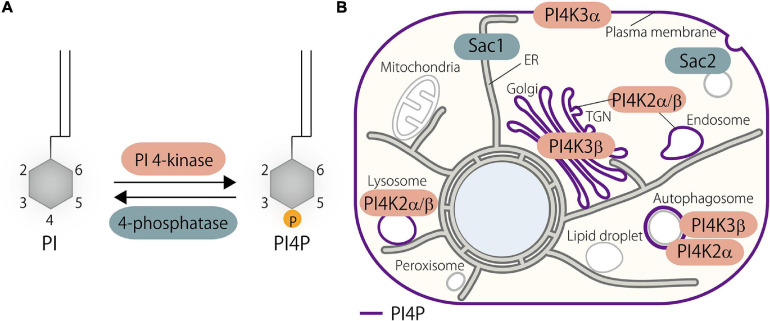
Phosphatidylinositol 4-phosphate (PI4P) metabolism in the cell. **(A)** *De novo* PI4P synthesis is catalyzed by PI 4-kinases that phosphorylate phosphatidylinositol (PI) at the D4 position of the inositol ring. Dephosphorylation of PI4P is mediated by 4-phosphatases. **(B)** Distribution of PI4P and its kinases and phosphatases in the mammalian cell. PI4P is synthesized at the plasma membrane (PM), Golgi, and endosomes/lysosomes by PI4K3α, PI4K3β, and PI4K2α/β, respectively. PI4K2α and possibly PI4K3β as well contribute to the generation of a pool of PI4P at autophagosomes. Metabolic degradation of PI4P is mediated by PI4P phosphatases such as Sac1 and Sac2/INPP5F. PI, phosphatidylinositol; PI4P, phosphatidylinositol 4-phosphate; TGN, *trans*-Golgi network.

PI4P, which is mono-phosphorylated at the 4-position of the inositol ring, is one of the most abundant phosphoinositides in eukaryotes. Its *de novo* synthesis is mediated by phosphatidylinositol 4-kinases (PI4Ks) that phosphorylate phosphatidylinositol (PI), the substrate, at the 4 position of the inositol ring (Balla, 2013). In mammals, there are four PI4Ks: two type III PI4Ks (PI4K3α and PI4K3β) and two type II PI4Ks (PI4K2α and PI4K2β) (Balla and Balla, 2006; Boura and Nencka, 2015). As a side note, type I PI4K turned out to be PI3K, and thus, no type I PI4Ks exists at present. The cellular distribution of PI4P is primarily determined by the localization as well as the site of action of its responsible kinases. PI4P is mainly distributed at the PM, the Golgi, and endosomes/lysosomes, and those pools of PI4P are synthesized by PI4K3α, PI4K3β, and PI4K2α or PI4K2β, respectively (Figure 1). A pool of PI4P at autophagosomes has also been demonstrated (Figure 1) (Albanesi et al., 2015; Wang et al., 2015; Judith et al., 2019; De Tito et al., 2020).

### Phosphatidylinositol 4-Kinases

PI4K3α localizes at the PM with the help of other regulatory proteins including EFR3A, EFR3B, TTC7A, TTC7B, FAM126A, FAM126B, and TMEM150A (Nakatsu et al., 2012; Baskin et al., 2015; Bojjireddy et al., 2015; Chung et al., 2015a). Biochemical as well as genetic evidences show that PI4K3α is required for PI4P production at the PM, and other PI4Ks are unable to compensate for this kinase, suggesting a distinct and non-overlapping function of PI4Ks (Nakatsu et al., 2012). PI4K3β localizes at the Golgi where it produces PI4P (Wong et al., 1997; Antonietta De Matteis et al., 2005). Several proteins, including Arf1 (Godi et al., 1999), ACBD3 (Sasaki et al., 2011) and PKD (Hausser et al., 2005), are reported to support its Golgi localization and function. Both type II PI4Ks, PI4K2α and PI4K2β, localize at endosomes or late endosomes/lysosomes and produce a pool of PI4P at those membranes (Balla and Balla, 2006). PI4K2α and PI4K2β localize at the endosomal membranes *via* palmitoylation (Balla et al., 2002; Barylko et al., 2009; Lu et al., 2012). PI4K2α has been shown to localize and generate a pool of PI4P at the *trans*-Golgi network (TGN) as well (Wang et al., 2003). PI4K2α and PI4K3β have been shown to associate with the autophagosomes, where they contribute to the generation of the pool of PI4P (Albanesi et al., 2015; Wang et al., 2015; Judith et al., 2019; De Tito et al., 2020).

PI4Ks were reported to localize in the nucleus and generate a pool of PI4P (reviewed in Chen et al., 2020). Recent findings have revealed the presence of PI, the precursor of PI4P, at the outer membrane of the mitochondria (Pemberton et al., 2020b; Zewe et al., 2020). In addition, functional involvement and localization of the TGN-derived vesicles containing PI4P, which is synthesized by PI4K3β, have been observed at the ER–mitochondria MCSs (Nagashima et al., 2020). However, no direct evidence for the existence of PI4P or PI4Ks at the mitochondria has been reported.

### Phosphatidylinositol 4-Phosphate Phosphatases

Metabolic degradation of PI4P (i.e., dephosphorylation) is controlled by the suppressor of actin (Sac)-domain containing phosphoinositide phosphatase family. The Sac phosphatase domain family in mammals consists of five members including Sac1, Sac2/INPP5F, Sac3/Fig4, Synaptojanin1, and Synaptojanin2, which all contain the Sac domain, a phosphoinositide phosphatase domain (Hsu and Mao, 2013). Sac1 is the major phosphatase that controls PI4P metabolism in the cell (Del Bel and Brill, 2018), although it dephosphorylates PI3P and PI(3,5)P_2_ in addition to PI4P (Guo et al., 1999; Nemoto et al., 2001). Sac1 is a type II transmembrane protein that localizes at the ER, but translocates to Golgi via COPII-mediated transport at the nutrient-limiting condition (Blagoveshchenskaya et al., 2008). Several pieces of evidence suggest that Sac1 dephosphorylates PI4P on the ER membranes and that it is transported from other membranes to the ER via MCSs (see below). This “in cis” action of Sac1 keeps the levels of PI4P low at the ER and, thus, critically contributes to the ORP-mediated lipid countertransport by creating a concentration gradient of this lipid between the ER and other membranes, which will be described later in detail. However, the “in trans” action of Sac1, in which it dephosphorylates PI4P on the PM or the Golgi membranes, was also reported (Stefan et al., 2011; Dickson et al., 2016; Venditti et al., 2019a).

Sac2/INPP5F and synaptojanins contribute to the metabolism of a pool of PI4P in the endocytic pathway. Synaptojanins have a 5-phosphatase domain that dephosphorylates PI(4,5)P_2_ in addition to the Sac1 domain (McPherson et al., 1996). The well-known site of action of synaptojanins is at the clathrin-coated pits. Synaptojanins are recruited to the clathrin-coated pits where they sequentially dephosphorylate PI(4,5)P_2_-PI4P-PI via 5-phosphatase and 4-phosphatase enzymatic activities (Mani et al., 2007; Cao et al., 2017). Likewise, Sac2/INPP5F is also recruited to the late phase of endocytic structures (Hsu et al., 2015; Nakatsu et al., 2015; Levin et al., 2017). Sac2/INPP5F interacts with OCRL, a 5-phosphatase that is also recruited to clathrin-coated pits (Pirruccello and De Camilli, 2012). OCRL has a 5-phosphatase domain but lacks a 4-phosphatase domain. Thus, OCRL dephosphorylates PI(4,5)P_2_ to PI4P, and then Sac2/INPP5F dephosphorylates PI4P to PI during endocytosis. Thus, OCRL and Sac2/INPP5F function as a split-synaptojanin to cooperatively dephosphorylate PI(4,5)P_2_ to PI (Nakatsu et al., 2015). A recent finding suggests a role of Sac2/INPP5F in the exocytic pathway (Nguyen et al., 2019).

## ORP Family Proteins

**FIGURE 2:**
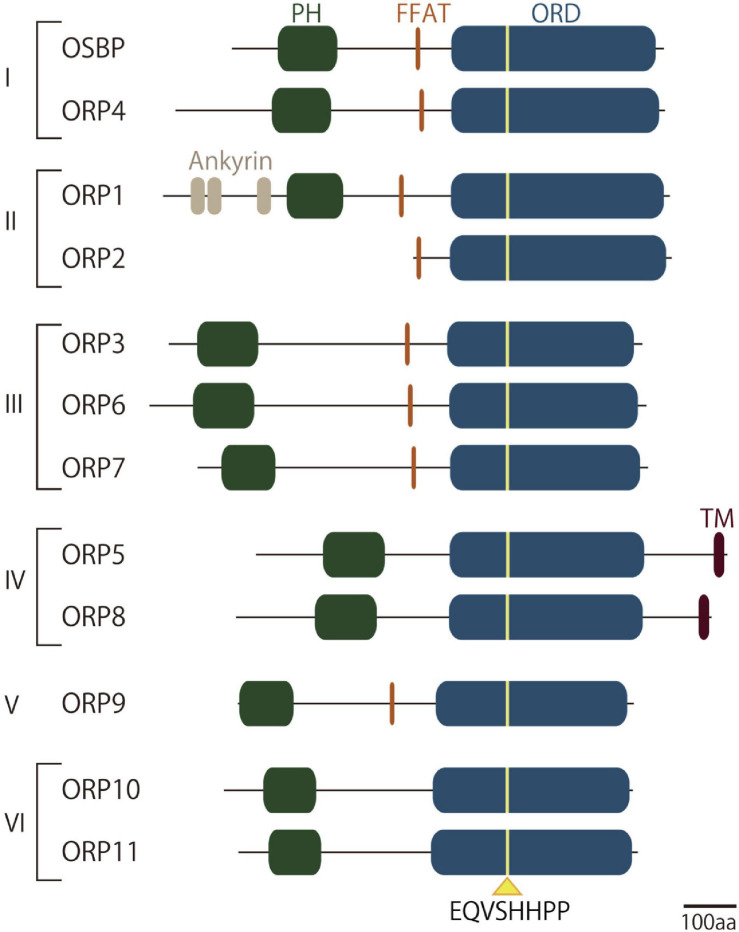
Domain structures of oxysterol-binding protein-related protein (ORP) family proteins. Schematic cartoon representing the domain structures of ORP family proteins in mammals. ORPs in mammals are subdivided into six groups according to their sequence homology and domain organization. They have a well-conserved lipid-binding domain called oxysterol-binding protein-related domain (ORD), in which a consensus lipid-binding motif EQVSHHPP is located. Most but not all of ORPs possess a PH domain that binds phosphoinositides such as PI4P and/or PI(4,5)P_2_ in the target membranes. Endoplasmic reticulum (ER) targeting determinants such as the FFAT (two phenylalanines in acidic tract)-motif, which is recognized by ER-resident membrane protein VAPA or VAPB, or membrane anchoring domain facilitates the localization of ORPs at membrane contact sites (MCSs) between the ER and other membranes. ORD, oxysterol-binding protein-related domain; FFAT, two phenylalanines in acidic tract.

ORPs are a family of LTPs that are highly conserved in eukaryotes (Olkkonen, 2015; Pietrangelo and Ridgway, 2018). Seven members in yeast and 12 members in humans have been identified, suggesting a requirement for multiple players that cover diverse cellular functions (Lehto et al., 2001; Raychaudhuri and Prinz, 2010). The 12 known ORPs in mammals are subdivided into six groups according to their sequence homology and domain organization: OSBP and ORP4 in group I, ORP1 and ORP2 in group II, ORP3, ORP6, and ORP7 in group III, ORP5 and ORP8 in group IV, ORP9 in group V, and ORP10 and ORP11 in group VI (Figure 2). Mounting evidence demonstrates that ORPs regulate a variety of cellular functions including, but not limited to, lipid transport, membrane/organelle trafficking, and signaling.

### Functional Domains in ORPs

#### Oxysterol-Binding Protein-Related Domain

Several domains or motifs are conserved in this family. One common feature of the ORP family members is the lipid-harboring domain called oxysterol-binding protein-related domain (ORD). Originally, OSBP, the first identified member of the ORP family, was identified as a cytosolic OSBP (Taylor et al., 1984). This study led to the discovery of the larger ORP family that commonly has an ORD as a lipid-transfer or lipid-sensing domain. Subsequent studies of OSBP as well as other ORPs revealed that the ORD accommodates not only oxysterols but also other lipids (Raychaudhuri and Prinz, 2010; de Saint-Jean et al., 2011; Maeda et al., 2013; Olkkonen, 2015; Pietrangelo and Ridgway, 2018). Structural analysis of the ORD from Osh4, one of the well-characterized ORPs in yeast, revealed a β-barrel-like structure, containing a hydrophobic pocket that accommodates oxysterol or cholesterol, and a lid-like structure that closes the pocket (Im et al., 2005). The 3-hydroxylgroup of the sterol is positioned at the bottom of the pocket, and the side chain is covered by the N-terminal lid. To date, the lipid ligands for ORDs include oxysterols, cholesterol, and phospholipids such as phosphoinositides, phosphatidylserine (PS), and/or phosphatidylcholine (PC). ORDs from all ORPs contain the well-conserved residues EQVSHHPP, a consensus lipid-binding motif located near the entrance of the pocket. Given that the tandem histidine residues are responsible for the binding to the head group of PI4P, the ORP family might be structurally adapted to harbor PI4P as a common ligand (de Saint-Jean et al., 2011; Tong et al., 2013).

#### Membrane Targeting Domains/Motif

Most ORPs possess a Pleckstrin homology (PH) domain (Lemmon, 2008) in their N-terminal portion that mediates membrane association. Many of them have been characterized to have a high affinity to phosphoinositides such as PI4P and/or PI(4,5)P_2_. Since phosphoinositides are the critical determinants of cellular membrane identity (Di Paolo and De Camilli, 2006), recognition of such lipids by a PH domain is a key process for localization and, hence, MCS formation by ORPs. They also often have another determinant that associates with the ER. The FFAT (two phenylalanines in acidic tract) motif is recognized by ER-resident membrane protein vesicle-associated membrane protein (VAMP)-associated protein A or B (VAPA or VAPB) with their major sperm protein (MSP) domain (Loewen et al., 2003). The FFAT motif is present in many other LTPs that function at MCSs (Murphy and Levine, 2016). Another ER-associating structure is the membrane spanning domain in ORP5 and ORP8, which enables ER localization on its own. These ER-anchoring determinants help bridge the ER and target membranes at MCSs, where they mediate lipid transfer functions.

### Lipid Transfer Regulation by ORPs

A major function of ORPs is to transfer their lipid ligands between cellular membranes (Raychaudhuri and Prinz, 2010). Their lipid transfer activity has been extensively studied *in vitro* (Wong et al., 2017). Purified ORD protein has been shown to extract lipids from artificial liposomal membranes. When mixed with two different liposomes, the ORD is able to transfer lipids from one liposome to another (Pietrangelo and Ridgway, 2018). In the cellular context, most, but not all, ORPs have been shown to mediate lipid transfer between cellular membranes. The mode of lipid transfer is either shuttling between two different membrane compartments that have certain distance or direct transfer at MCSs (Wong et al., 2019).

**FIGURE 3:**
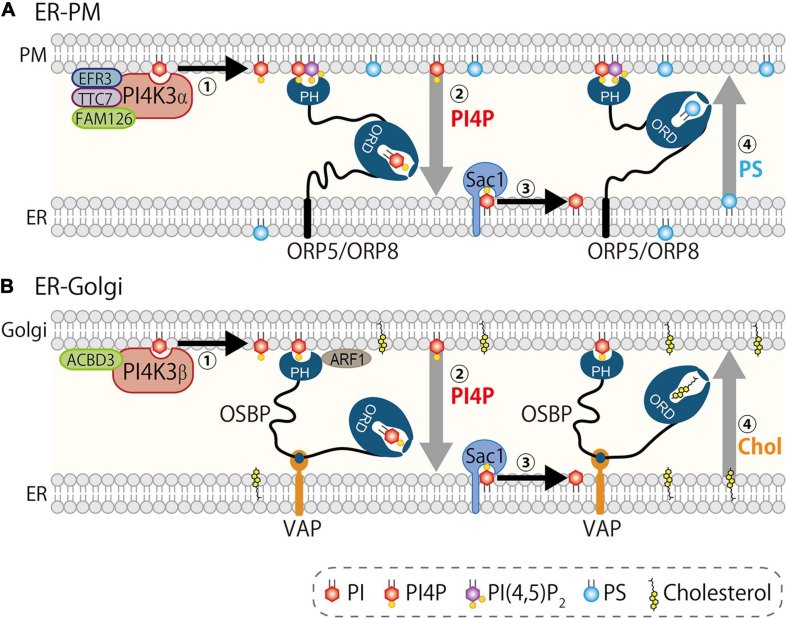
PI4P-driven lipid countertransport at MCSs. PI4P drives lipid countertransport mediated by ORP5/8 at ER–PM MCSs **(A)** and by OSBP at ER–Golgi MCSs **(B)**. **(A)** PI4K3α complex including EFR3A/B, TTC7A/B, and FAM126A/B synthesizes and concentrates PI4P at the PM (step 1). ORP5/8, both of which are anchored to the ER, form MCSs via interaction with PI4P and PI(4,5)P_2_ by PH domain, and transport PI4P (driver-ligand) from the PM to the ER (step 2). Sac1 dephosphorylates PI4P to PI, which keeps the concentration of PI4P low at the ER (step 3). ORP5/8 transport PS (cargo-ligand) from the ER to the PM. **(B)** PI4K3β generates PI4P upon recruitment to the Golgi by regulatory proteins including Arf1 (step 1). Oxysterol-binding protein (OSBP) is recruited to the ER–Golgi MCSs *via* PH domain that interacts with PI4P and ACBD3 and/or Arf1 on the Golgi membranes and FFAT-motif that binds vesicle-associated membrane protein (VAMP)-associated protein A/B (VAPA/B). Then, OSBP mediates transport of PI4P (driver-ligand) from the Golgi to the ER (step 2). Sac1 hydrolyzes PI4P to PI (step 3). OSBP transports cholesterol (cargo-ligand) to Golgi (step 4). Synthesis and hydrolysis of PI4P by PI4Ks and Sac1 establishes a concentration gradient of this lipid between the ER and the PM/Golgi, which determines the flow of driver-ligand PI4P to the ER and counterflow of cargo-ligands from the ER.

An interesting nature of ORPs is their lipid exchange activity. OSBP or its yeast counterpart Osh4/Kes1 was initially demonstrated to be a sterol transfer protein (Raychaudhuri et al., 2006; Ngo and Ridgway, 2009). Indeed, they transfer cholesterol (or ergosterol in yeast) between membranes both *in vitro* and in live yeast. However, de Saint-Jean et al. (2011) elegantly demonstrated that Osh4/Kes1 transfers not only sterol, but also PI4P via its ORD. Its crystal structure showed that the ORD of Osh4/Kes1 accommodates either sterols (cholesterol, ergosterol, and oxysterols) or PI4P. An interesting point was that Osh4/Kes1 mediates exchange of sterol and PI4P between two different liposomes containing either lipid. Subsequently, the same group extended this idea to demonstrate that OSBP mediates countertransport of PI4P and cholesterol at MCSs between the ER and Golgi (Mesmin et al., 2013). ORP5 and ORP8 have also been demonstrated to mediate countertransport of PI4P and PS at ER–PM MCSs by our group (Chung et al., 2015b) (Figure 3). In this lipid countertransport, differing amounts of PI4P between the ER and other membranes such as Golgi or PM is the driving force. PI4P is continuously synthesized at the PM or Golgi by PI4K3α or PI4K3β, respectively, while it is metabolically degraded at the ER by Sac1. This enzymatic regulation establishes a concentration gradient of PI4P between the ER and the PM or Golgi. Given that the ORD accommodates only one lipid molecule at a time (Im et al., 2005), the ORD picks up PI4P at the PM or Golgi and transfers it down a concentration gradient to the ER where Sac1 hydrolyzes it to PI. This PI4P flow empowers backward transfer of another lipid, PS for ORP5/8 or cholesterol for OSBP, from the ER to the PM or Golgi (Figure 3). This PI4P-driven lipid countertransport is further ensured by the mechanism by which these ORPs establish MCSs. OSBP or ORP5/8 forms MCSs via PH domain-mediated recognition of PI4P (with the help of other factors such as Arf1 or PI(4,5)P_2_ (see below for details), which guarantees the concentration gradient of this lipid at the MCSs. The PI4P metabolic cycle generated by its kinases and phosphatase is tightly coupled to the ORP function, and this functional partnership supports PI4P-driven lipid countertransport at MCSs by ORPs (Mesmin and Antonny, 2016).

## Lipid Transport by ORPs at Membrane Contact Sites

**FIGURE 4:**
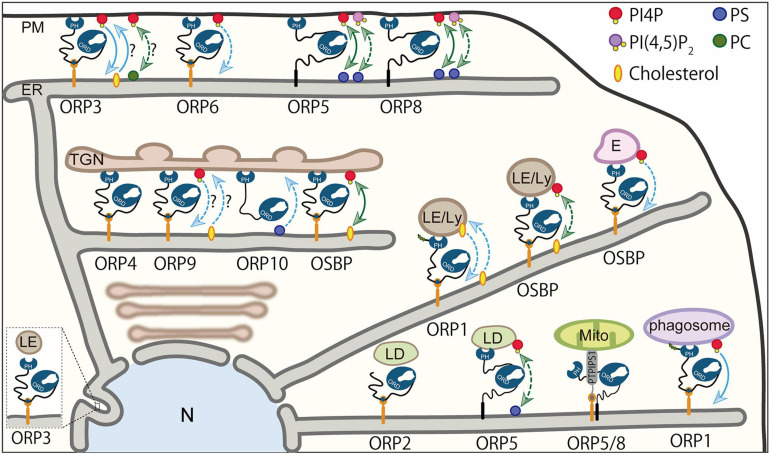
Lipid transport/countertransport mediated by ORPs at MCSs. Summary of ORPs mediating transport or countertransport of lipids at MCSs. At the ER–PM MCSs, ORP5 and ORP8 mediate countertransport of PI4P and PS driven by PI4P metabolic cycle. They have been proposed to act as PI(4,5)P_2_/PS exchangers. ORP3 and possibly ORP6 mediate PI4P transport to the ER, while ORP3 may transport PC or cholesterol from the ER in exchange for PI4P. At the ER–Golgi MCSs, OSBP is an exchanger of PI4P and cholesterol. ORP10 has been suggested to mediate PS transport from the ER. The function of ORP4 and ORP9 as transporters/exchangers is unknown. At the MCSs between the ER and endosomes, late endosomes, or lysosomes, OSBP functions as a PI4P transporter or PI4P/cholesterol exchanger. ORP1 acts as a sensor or transporter of cholesterol and a transporter of PI4P. At the ER–lipid droplet (LD) MCSs, ORP5 has been proposed to be a PI4P/PS exchanger. The role of ORP2 as a transporter/exchanger at the ER–LD MCSs is unknown. At the ER–mitochondria MCSs, ORP5 and ORP8 may or may not be exchangers/transporters for PI4P and PS. Blue arrows indicate “transport,” whereas green arrows show “countertransport.” Solid lines are used if the transport or countertransport of the indicated lipids has been demonstrated by loss-of-function or gain-of-function analysis in an acute manner (e.g., acute inhibition or manipulation of ORP proteins). Dashed lines are used if the transport or countertransport of the indicated lipids has been suggested by loss-of-function or gain-of-function analysis in a chronic manner [e.g., chronic inhibition (knock-down or knock-out) or overexpression of ORP proteins]. ER, endoplasmic reticulum; E, endosomes; LE/Ly, late endosomes/lysosomes; Mito, mitochondria; N, nucleus; PM, plasma membrane; TGN, *trans*-Golgi network.

The ER, the site of the synthesis of most of lipids, is now known to make MCSs with most of the organelles or the PM where ORPs mediate transport or countertransport of lipids. Accumulating evidence demonstrates that ORP family proteins are widely localized at distinct MCSs and operate their own lipid transport/countertransport function (Figure 4 and Table 1). Furthermore, the tight regulation of such ORP function by phosphoinositides has also become evident. This section provides an overview of the role of ORPs in lipid transport or exchange at MCSs and their contributions to cellular biological processes. How phosphoinositides, such as PI4P, regulate ORP function will also be discussed.

**TABLE 1:** Oxysterol-binding protein-related proteins (ORPs) that mediate transport or countertransport of lipids at membrane contact sites (MCSs).

ORPs	Lipid transfer activity *in vitro* ^A^	Localization at ER MCS (lipids transferred or exchanged)^B^	References
OSBP	PI4P/DHE	ER-Golgi **(PI4P/Sterol),** ER-endosome (PI4P), ER-LE/Ly (PI4P/Cholesterol)	Mesmin et al. (2013, 2017), Dong et al. (2016), Goto et al. (2016), Lim et al. (2019)
ORP1	DHE	ER-LE/Ly (Cholesterol), ER-phagosome **(PI4P)**	Eden et al. (2016), Zhao and Ridgway (2017), Dong et al. (2019), Levin-Konigsberg et al. (2019)
ORP2	PI(4,5)P_2_/DHE	ER-LD	Weber-Boyvat et al. (2015b)
ORP3	?	ER-PM **(PI4P)**	Weber-Boyvat et al. (2015a), D’Souza et al. (2020), Gulyás et al. (2020)
ORP4	Cholesterol	ER-Golgi	Wyles et al. (2007), Charman et al. (2014), Pietrangelo and Ridgway (2018)
ORP5	PI4P/PS, PI(4,5)P_2_/PS, DHE	ER-PM **(PI4P/PS),** ER-PM (PI(4, 5)P_2_/PS), ER-LD (PI4P/PS), ER-Mito	Chung et al. (2015b), Galmes et al. (2016), Ghai et al. (2017), Sohn et al. (2018)
ORP6	?	ER-PM (PI4P)	Mochizuki et al. (2018)
ORP7	?	?	
ORP8	PI4P/PS, PI(4,5)P_2_/PS	ER-PM **(PI4P/PS),** ER-PM (PI(4,5)P_2_/PS), ER-Mito	Chung et al. (2015b), Galmes et al. (2016), Ghai et al. (2017), Sohn et al. (2018)
ORP9	Cholesterol	ER-Golgi	Ngo and Ridgway (2009), Venditti et al. (2019b)
ORP10	?	ER-Golgi (PS)	Venditti et al. (2019b)
ORP11	?	?	

*^*A*^Lipids were transported or exchanged between liposomes *in vitro*. The exchanged lipids are separated by slash (/).*

*^*B*^Lipids demonstrated to be transported or exchanged in intact cells are shown in parentheses. The exchanged lipids are separated by slash (/).*

*^*B*^Lipids shown in **bold** font: transport or exchange of the indicated lipids has been demonstrated in loss-of-function or gain-of-function analysis in an acute manner (e.g., acute inhibition or manipulation of ORP proteins).*

*^*B*^Lipids shown in regular font: transport or exchange of the indicated lipids has been suggested in loss-of-function or gain-of-function analysis in a chronic manner [e.g., chronic inhibition (knock-down or knock-out) or overexpression of ORP proteins].*

*?, Unknown.*

### Endoplasmic Reticulum–Plasma Membrane Membrane Contact Sites

#### ORP5/8

ORP5 and ORP8, which belong to group IV of the ORP family, have similar characteristics of domain structures (Figure 2). Both proteins have a PH domain, a coiled-coil domain, a linker region, an ORD, and a membrane-spanning domain. Unlike other ORPs, ORP5 and ORP8 anchor to the ER *via* a membrane-spanning domain located at the C-terminus. The PH domain of ORP5 or ORP8 recognizes PI4P and/or PI(4,5)P_2_ in the PM with different preferences, thereby making an MCS between the ER and the PM (Chung et al., 2015b; Ghai et al., 2017; Sohn et al., 2018). It has been demonstrated that the ORD of ORP5/8 specifically harbors PI4P or PS and transfers them between liposomes *in vitro*. Intriguingly, the transfer of PS from donor to acceptor liposomes was strongly enhanced if another lipid ligand PI4P was present in the acceptor side, and the opposite combination also showed the same tendency, indicating an exchange activity of the ORP8 ORD (Chung et al., 2015b). In addition to PI4P and PS, the ORP5 ORD was shown to transport dehydroergosterol (DHE) *in vitro*, and this DHE transport was partially inhibited in the presence of PI4P in the donor liposomes (Du et al., 2011) (see the section “Endoplasmic Reticulum–Endosome/Lysosome/Autophagosome/Phagosome Membrane Contact Cites” for more details). In the cellular context, ORP5/8 exchanges PS with PI4P between the ER and the PM. Functional ablation of PI4K3α (the PI4P supplier at the PM) or Sac1 (the PI4P remover at the ER) disrupted the countertransport of those lipids, confirming that ORP5/8-mediated lipid countertransport is tightly coupled to the PI4P metabolic flow between the PM and the ER (Chung et al., 2015b) (Figure 3). This ORP5/8-mediated PI4P-driven lipid countertransport enables PS supply from the ER to the PM against its concentration gradient. Similarly, PI4P-driven PS transport at ER–PM MCSs has also been demonstrated in yeast (Moser von Filseck et al., 2015).

**FIGURE 5:**
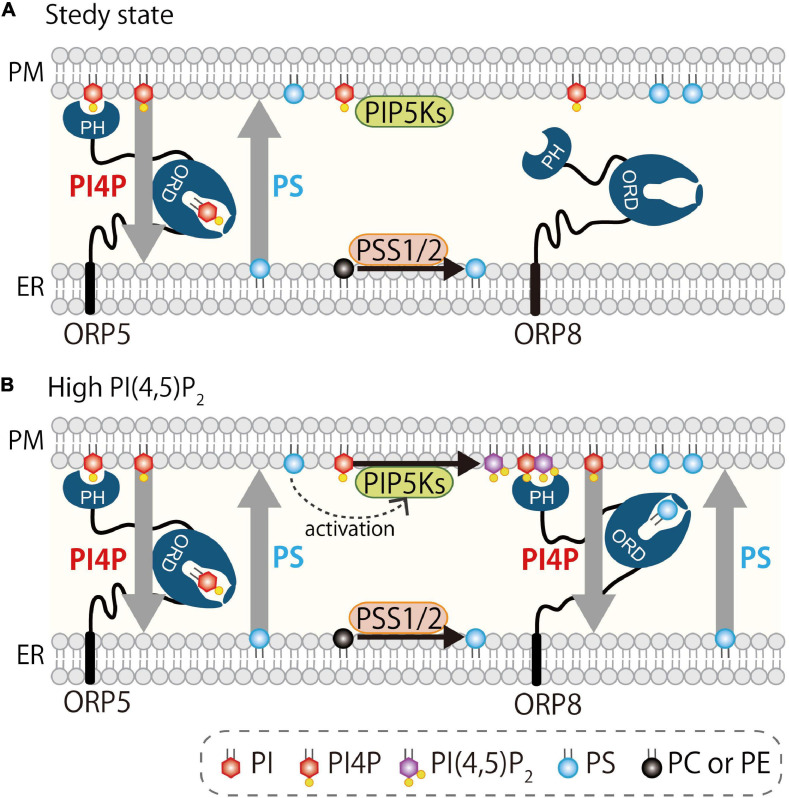
Regulation of PI4P and PI(4,5)P_2_ at the PM by oxysterol-binding protein-related proteins 5 and 8. **(A)** At steady state, ORP5 localizes at the ER–PM MCSs *via* binding to PI4P and PI(4,5)P_2_. **(B)** When PI(4,5)P_2_ is high at the PM, ORP8 is strongly recruited to the PM via preferential binding to PI(4,5)P_2_ and mediates countertransport of PI4P and PS. This reduces PI4P levels at the PM and thus contributes indirectly to reducing PI(4,5)P_2_ levels by limiting its precursor. ORP8-mediated countertransport of PS to the PM may facilitate the activation of PIPKs (Nishimura et al., 2019).

Several studies showed the regulation of PM PI(4,5)P_2_ by ORP5 and ORP8. Results from Sohn et al. (2018) support the role of ORP5/8 in exchanging PS with PI4P at ER–PM MCSs. In this study, BRET imaging quantitative assay was used to show that ORP5/8 controls PI(4,5)P_2_ levels by tuning the amount of its precursor PI4P, and this is basically controlled by their localization to the MCSs via the PH domain. The ORP5 PH domain requires both PI4P and PI(4,5)P_2_ for localization at ER–PM MCSs. However, the ORP8 PH domain strongly depends on PI(4,5)P_2_ for its MCS localization, although PI4P is still required even when PI(4,5)P_2_ production is increased. In the situation where PI(4,5)P_2_ is highly produced at the PM, PI4P levels become low due to ORP8 localization at the MCSs, thereby limiting PI4P availability for PI(4,5)P_2_ conversion by PIPKs. They proposed that this is a rheostat mechanism for tightly controlling the PI(4,5)P_2_ levels in a narrow range (Figure 5). ORP5 might be a housekeeper for PI4P and PI(4,5)P_2_ (and PS) homeostasis at the PM in the steady-state condition, while ORP8 could be a regulator for more stimulatory situations that might require tight regulation of PI(4,5)P_2_ (e.g., Ca^2+^ regulation, cell migration, receptor activation, or membrane ruffling).

In contrast, another study by the Yang group (Ghai et al., 2017) proposed a different model for the regulation of PI(4,5)P_2_ by ORP5 and ORP8. They demonstrated that localization of ORP5 and ORP8 is dependent on PI(4,5)P_2_, but not on PI4P, and this is due to the binding property of their PH domains to PI(4,5)P_2_, but not to PI4P. Furthermore, the driver-ligand of the ORP5/8 ORD was proposed to be PI(4,5)P_2_. In an *in vitro* lipid transport assay, they showed that the ORP8 ORD efficiently transports PI(4,5)P_2_, and its concentration gradient between two liposomes enhanced the PS exchange. Consistent with this idea, PM PI(4,5)P_2_ levels increased by knockdown of ORP5/8. These authors proposed that ORP5 and ORP8 are PI(4,5)P_2_/PS exchangers in a PI(4,5)P_2_-driven mechanism. Their conclusion regarding the role of ORP5 and ORP8 in controlling PI(4,5)P_2_ levels at the PM agreed with that of the Balla group, but the underlying mechanism differed. Because of the efficient *in vitro* PI(4,5)P_2_ transfer activity in addition to a strong dependency on PI(4,5)P_2_ for PM localization, the localization and function of ORP5/8 appear to be PI4P-independent. In the PI4P-driven model (Figure 3), PI4P is transported from the PM to the ER where it is hydrolyzed by the PI4P phosphatase Sac1, and this PI4P metabolic cycle generates the PI4P concentration gradient that drives lipid countertransport by ORP5 and ORP8 (Chung et al., 2015b). However, in the case of the PI(4,5)P_2_-driven model, how PI(4,5)P_2_ is metabolically degraded at the ER to create the concentration gradient of this lipid is still unknown. The authors mentioned that INPP5E might be involved in this process, but there is no evidence showing that INPP5E localizes at the ER and hydrolyzes PI(4,5)P_2_ there. Instead, INPP5K, an ER-localized 5-phosphatase reported to hydrolyze PI(4,5)P_2_ and PI(3,4,5)P_3_, is a candidate, although the site of action of INPP5K has been proposed to be not only the ER, but also other membranes, including the PM, nucleus, and autolysosomes (Gurung et al., 2003; Ijuin and Takenawa, 2003; Hung et al., 2009; Dong et al., 2018; Ramos et al., 2020; McGrath et al., 2021).

ORP5/8-mediated countertransport and its relationship with the PI4P–PI(4,5)P_2_–PS metabolic axis was further reported. A recent yeast study (Nishimura et al., 2019) showed that an osh-mediated lipid exchange mechanism generates a local domain containing unsaturated PS and sterol that promotes the localization and activation of PIPK, leading to PI(4,5)P_2_ production. This study further points to the functional relationship between PI4P/PS exchange and PI(4,5)P_2_ regulation. Additionally, a relationship between PS metabolism and MCS formation was also reported (Sohn et al., 2016) in studies of PSS1 (Kuge et al., 1991) and PSS2 (Kuge et al., 1997), the two PS synthases whose genetic mutation leads to Lenz–Majewski syndrome (Lenz and Majewski, 1974). The ER-localized enzyme PSS1 or PSS2 catalyzes PS production using PC or phosphatidylethanolamine (PE), respectively, as a substrate. Their enzyme activity was shown to be inhibited by the end product PS, and the mutation responsible for this feedback inhibition, which caused Lenz–Majewski syndrome, leads to PS accumulation in the ER (Sousa et al., 2014). Inhibition of PI4K3α by the specific inhibitor A1 decreases the PM PI4P levels, and this results in the inhibition of PS synthesis by approximately 50%. This is likely a homeostatic regulation because PS synthesis needs to be slowed down in a situation where PI4P-driven PS transport is slow. In fact, expression of the PSS1 disease mutant, which reflects a disease condition where PS accumulated at the ER, led to a reduction of ORP8 membrane association as well as a decrease in PM PI4P levels (Sohn et al., 2016). Collectively, PI4P-driven lipid countertransport mediated by ORP5 and ORP8 is tightly coupled to homeostasis of lipids such as PI4P, PI(4,5)P_2_, and PS at the PM and the ER (Balla et al., 2019, 2020; Pemberton et al., 2020a; Santos et al., 2020) (Figure 5).

#### ORP3

ORP3 is categorized in group III, together with ORP6 and ORP7 (Figure 2). ORP3 contains a typical cytosolic ORP family domain architecture, such as a PH domain, FFAT-motif, and ORD. ORP3 is basically cytosolic at steady state, but translocates to the ER–PM MCSs upon PKC activation and Ca^2+^ influx (Weber-Boyvat et al., 2015a; Gulyás et al., 2020). ORP3 associates with the ER via FFAT motif-mediated binding to VAP, and it appears to be phosphorylation dependent (Lehto et al., 2005; Weber-Boyvat et al., 2015a). The PM association of ORP3 is mediated by its PH domain that recognizes PI4P and PI(4,5)P_2_ (Gulyás et al., 2020). Such phosphorylation-dependent binding of ORP3 to VAP and the PM might imply that phosphorylation may induce a conformational change that unmasks the PH domain and FFAT-motif. Colocalization of ORP3 with ORP6 or ORP8 at the ER–PM MCSs has been reported (Weber-Boyvat et al., 2015a; Mochizuki et al., 2018). ORP3-mediated ER–PM MCS formation has been linked to cellular processes such as Ca^2+^ regulation, adhesion, and migration (Machaca, 2020). A recent study (D’Souza et al., 2020) provided a mechanistic insight into how ORP3 controls focal adhesion dynamics. Those studies indicate the following scenario. Store-operated Ca^2+^entry (SOCE) by STIM1-Orai1 axis activates PKC and then induces the ORP3 translocation to the ER–PM MCSs where STIM1 and Orai1 also localize. This ORP3 translocation occurs around the focal adhesion where ORP3 recruits the guanin exchange factor IQSec1 that activates Arf5, thereby promoting the disassembly of focal adhesion at the rear front of the cell. How ORP3-mediated lipid transport/countertransport is involved in those processes, however, is still unclear. This is because the ligand(s) of the ORD have not been firmly identified. However, an imaging study showing a strong reduction of PI4P, but not of PI(4,5)P_2_, PI(3,4,5)P_3_, or PS, at the PM after acute recruitment of ORP3 to the ER–PM MCS, suggests PI4P as a ligand of the ORP3 ORD (Gulyás et al., 2020). Other cargo-ligand(s) could be PC or cholesterol (D’Souza et al., 2020; Gulyás et al., 2020). However, whether ORP3 is indeed an exchanger of those candidate ligands has not been clearly demonstrated and, thus, needs further investigation.

#### ORP6

ORP6 is another member in group III (Figure 2). Like ORP3, ORP6 also shows a typical domain architecture such as N-terminal PH domain, FFAT-motif, and ORD. Mochizuki et al. (2018) demonstrated that ORP6 colocalized with ORP3 or extended synaptotagmins (E-Syts), but not with ORP5, at the ER–PM MCSs in neuronal cells such as Neuro2A or primary cerebellar granule cells. The ORP6 PH domain binds PI4P, PI(4,5)P_2_, and phosphatidic acid (PA) in a membrane lipid strip assay. ORP6 knockdown led to an increase in PM PI4P detected by the OSBP PH domain probe, suggesting that ORP6 contributes to the PI4P turnover at the PM. However, whether ORP6 mediates transport or countertransport of lipids is still unclear, as well as its contribution to cell physiology.

### Endoplasmic Reticulum–Golgi Membrane Contact Sites

#### OSBP

OSBP is the founding member of the ORP family (Raychaudhuri and Prinz, 2010) (Figure 2). The domain architecture is PH domain, FFAT-motif, and ORD, the typical ORP family domain structure. OSBP represents both a cytosolic pool and membrane-bound status. Initially, OSBP was shown to associate with the Golgi including the TGN, but also with endosomes or lysosomes in later studies. The membrane targeting of OSBP is mediated by PH domain as is the case for other ORPs. According to an *in vitro* liposome-binding assay, the OSBP PH binds PI4P and PI(4,5)P_2_ (Rameh et al., 1997; Levine and Munro, 1998). However, a study using yeast as a model (Levine and Munro, 2002) showed the OSBP PH domain localizes in the Golgi, and this Golgi localization was abolished upon deletion of the PI4-kinase *pik1* (the yeast ortholog of mammalian PI4K3β that synthesizes PI4P at the Golgi). The deletion of the PIP-kinase *mss4* [the yeast ortholog of mammalian PIPKs that generates PI(4,5)P_2_], however, did not abolish the Golgi localization of the OSBP PH domain, indicating that PI4P is the key to Golgi localization *in situ*. Furthermore, the OSBP PH domain also interacted with the GTP-bound form of Arf1, the small GTPase that controls membrane association of Golgi proteins (Levine and Munro, 2002). Arf1 also controls recruitment to the Golgi of PI4K3β, the PI4K responsible for the synthesis of a pool of PI4P at this organelle (Godi et al., 1999). PI4K2α, another PI4K that synthesizes PI4P at endosomes and the TGN (Wang et al., 2003), is also shown to provide a pool of PI4P at the TGN for OSBP recruitment (Mesmin et al., 2017). Thus, the OSBP PH domain recognizes both PI4P and GTP-Arf1. This coincident detection mechanism ensures the targeting of OSBP to the Golgi, and thus, OSBP localizes at ER–Golgi MCSs with the FFAT motif captured by VAP at the ER.

The OSBP ORD has been demonstrated to exchange PI4P and cholesterol between the ER and Golgi (Mesmin et al., 2013). This idea, basically, came from a study by de Saint-Jean et al. (2011) using osh4 as a model. Extraction of fluorescent ergosterol DHE by Osh4p, which was previously shown to bind sterol in its ORD, was inhibited by PI4P, but not by many other lipids tested. This was due to the surprising ability of the osh4 ORD to solubilize PI4P by itself. In fact, crystal structural analysis clearly revealed that osh4 specifically harbors PI4P or cholesterol in its ORD. The acyl chain of PI4P is inserted deep inside the pocket, and the head group of PI4P is positioned near the entrance that contains the conserved sequence containing tandem histidines. Additionally, a series of elegant *in vitro* lipid transport experiments demonstrated that osh4 exchanges sterol with PI4P between liposomes (de Saint-Jean et al., 2011).

The study above led to the discovery of OSBP function at ER–Golgi MCSs. Mesmin et al. (2013) demonstrated that OSBP exchanges cholesterol and PI4P at ER–Golgi MCSs. Mechanistically, OSBP extracts PI4P from the Golgi membranes and transfers it to the ER, and this PI4P flow along its gradient ensures the back transfer of cholesterol against the gradient by OSBP (Figure 3). Functionally, OSBP has been estimated to mediate one-third to two-thirds of cholesterol transport by consuming approximately half of the total cellular PI4P, according to an acute pharmacological inhibition study (Mesmin et al., 2017) using the chemical OSW-1 (Burgett et al., 2011). Such inhibition of OSBP led to a roughly fourfold increase in PI4P levels at the TGN and a roughly twofold increase in whole cells. A recent study using a different inhibitor also reported a similar effect (Péresse et al., 2020). These data suggest the physiological contribution of OSBP in the regulation of PI4P and cholesterol at the TGN. However, another study demonstrated no major impact on PI4P levels at the TGN by OSBP knockdown or addition of 25-hydroxycholesterol (Goto et al., 2016). Chronic inhibition by knockdown (in contrast to acute inhibition) as well as a cell type difference might be the reasons for the apparently different results. Regarding the 25-hydroxycholesterol, no inhibitory (but even a slight stimulatory) effect on the OSBP-mediated PI4P transport between liposomes has been demonstrated (Mesmin et al., 2017). This could be a possible explanation for the very minor effect of this lipid on the PI4P levels in the TGN. Collectively, OSBP regulates PI4P levels at the TGN.

#### ORP4

ORP4 belongs to group I, together with OSBP (Figure 2). ORP4 has a PH domain, FFAT motif, and ORD, and there is a short isoform containing only an ORD. ORP4 has been detected in the brain, kidneys, heart, skeletal muscles, and spleen by Northern blot analysis of human tissues (Wang et al., 2002), as well as in the brain and testis by Western blot analysis of mouse tissues (Udagawa et al., 2014). The PH domain bound to PI4P in a membrane lipid strip assay, as well as in a liposome-binding assay (Charman et al., 2014). In CHO cells, the PH domain of ORP4 weakly associated with the Golgi. The purified protein containing the ORP4 ORD binds 25-hydroxycholesterol to extract and transfer cholesterol between liposomes (Charman et al., 2014). ORP4 was shown to interact with OSBP and localizes to the Golgi in an OSBP-dependent manner, suggesting that ORP4 functions at the ER–Golgi MCSs with OSBP (Wyles et al., 2007; Pietrangelo and Ridgway, 2018). However, whether ORP4 mediates transport or countertransport of PI4P and/or cholesterol is unknown. ORP4 has been implicated in several cancers, including leukemia, as a signaling regulator; however, its role as an LTP in MCSs is unclear (Fournier et al., 1999; Silva et al., 2001; Henriques Silva et al., 2003; Zhong et al., 2016).

#### ORP9

ORP9 is the sole member in group V (Figure 2). The domain structure of the full-length long form is a typical one containing a PH domain, FFAT motif, and ORD. The short isoform lacking the PH domain has also been reported. The ORP9 PH domain binds mono-phosphorylated phosphoinositides according to a lipid membrane overlay assay and cosediments with liposomes containing PI4P (Ngo and Ridgway, 2009). Purified full-length ORP9 proteins extract cholesterol and PI4P, but not oxysterol or PS, from liposomes *in vitro* and transfer cholesterol between liposomes (Ngo and Ridgway, 2009; Liu and Ridgway, 2014). This cholesterol transfer activity is enhanced if the donor liposomes also contain PI4P, and this enhancement is dependent on its PH domain, suggesting that such enhancement is due to the efficient targeting of purified ORP9 proteins to the donor liposomes by PH domain (Ngo and Ridgway, 2009). Whether ORP9 exchanges PI4P and cholesterol is still unknown. ORP9 localizes partially at the TGN and does not colocalize with PI4K3β. ORP9 knockdown in HeLa cells did not alter the PI4P levels in the TGN, as assessed by immunofluorescence staining with anti-PI4P antibody (Liu and Ridgway, 2014). A recent study demonstrated a role of ORP9 in the integrity of ER–TGN MCSs as its depletion in addition to simultaneous depletion of OSBP affects the formation of ER–TGN MCSs assessed by FLIM (fluorescence lifetime imaging) (Venditti et al., 2019b).

#### ORP10

ORP10 is a member of group VI, and it has a PH domain and an ORD, but lacks a FFAT motif (Figure 2). ORP10 is reported to localize at the Golgi *via* its PH domain. The ORD of ORP10 has been shown to extract PS from liposomes (Maeda et al., 2013). Venditti et al. (2019b) demonstrated that ORP10 localized at the MCSs between the ER and TGN. ORP10 depletion in HeLa cells affects the integrity of ER–TGN MCSs and leads to reduced PS levels in the Golgi, suggesting its role as a PS transporter. The residues involved in binding to PI4P and PS in the ORP5/8 ORD are well conserved in ORP10, and mutations in these residues were shown to abolish the ability to rescue the integrity of the MCSs in ORP10-deficient cells. These results suggest that ORP10 might be a lipid exchanger. However, a lipid exchange function of ORP10 has not been demonstrated.

### Endoplasmic Reticulum–Endosome/Lysosome/Autophagosome/Phagosome Membrane Contact Sites

#### OSBP

OSBP has been reported to localize at MCSs other than the Golgi. Dong et al. (2016) demonstrated the function of OSBP at ER–endosome MCSs. OSBP knockdown as well as VAPA/VAPB deletion leads to endosomal PI4P accumulation and then actin reorganization such as the loss of stress fibers and WASH-dependent actin comet formation. OSBP-mediated transport of PI4P from endosomes to the ER contributes to the negative regulation of PI4P at endosomes. Sobajima et al. (2018) showed the function of OSBP at the MCSs between endosomes and the TGN, but not the ER. OSBP interacts with RELCH, a novel Rab11-GTP effector, and tethers recycling endosomes to the TGN by the OSBP–RELCH-Rab11 complex. This complex meditates the transfer of cholesterol from recycling endosomes to the TGN (Sobajima et al., 2018). Another study (Lim et al., 2019) also indicates the role of OSBP-mediated cholesterol transport at ER–lysosome MCSs. OSBP was found to supply cholesterol to lysosomes *via* ER–lysosome MCSs. This pool of cholesterol in the limiting membrane of lysosomes triggers the activation of mTORC1, the master regulator of growth, via Rag GTPases as well as the amino acid permease SLC38A9 (Castellano et al., 2017). In fact, OSBP inhibition by the chemical inhibitor OSW1 or shRNA-mediated knockdown reduced cholesterol accumulation on the lysosomal-limiting membranes in cells lacking Niemann Pick C type 1 (NPC1), thereby suppressing the hyperactivation of mTORC1 (Lim et al., 2019). The studies described above all indicate an important role of OSBP as a transporter, but not as a *bona fide* exchanger, of lipid ligands at several MCSs. Despite the fact that OSBP is an exchanger of PI4P and cholesterol at the ER and Golgi, whether and how such exchange activity of OSBP support those functions at the MCSs other than Golgi is currently unclear.

#### ORP5

ORP5 has been suggested to functionally contribute to the cholesterol transport from late endosomes/lysosomes to the ER (Du et al., 2011). Purified ORP5 ORD mediates transfer of DHE between liposomes, and this activity is partially inhibited by PI4P, but not by PI3P, PI5P, or PI(4,5)P_2_, suggesting a possibility of ORP5 as a cholesterol transporter. Transiently expressed full-length ORP5 or its ORD was co-immunoprecipitated with either exogenously expressed or endogenous NPC1. Their association might be direct or indirect. Knockdown of ORP5, but not of ORP8, resulted in accumulation of cholesterol in the limiting membrane of late endosomes/lysosomes and impairment of cholesterol transfer from those organelles to the ER (evaluated by ACAT-mediated cholesterol esterification at the ER). The authors suggest that ORP5 may function with NPC1 as a cholesterol transporter *via* MCSs between the ER and lysosomes, although such MCS formation has not been demonstrated to date.

#### ORP1

ORP1, which belongs to group II together with ORP2, exists in long (L) and short (S) forms (Figure 2). ORP1L contains ankyrin repeats in addition to other typical domains for ORPs such as a PH domain, FFAT-motif, and ORD. ORP1S encodes only an ORD but lacks other domains. Purified PH domain of ORP1L binds weakly PI(3,4)P_2_, PI(3,5)P_2_, and PI(3,4,5)P_3_ in liposome-binding assay (Johansson et al., 2005). ORP1L binds Rab7 *via* ankyrin repeats (Johansson et al., 2005) as well as VAP *via* FFAT motif and, hence, localizes at the MCSs between the ER and late endosomes/lysosomes or autophagosomes. The ORP1 ORD binds oxysterol, cholesterol, and PI4P (Vihervaara et al., 2011; Zhao and Ridgway, 2017; Zhao et al., 2020). Dong et al. (2019) reported that the ORD binds all of the phosphoinositides with a similar extent compared with DHE but does not bind PS. Lipid transfer activity of the ORP1 ORD has also been demonstrated (see below). However, its exchange activity has not been reported to date.

#### Cholesterol Transport by ORP1

Dong et al. (2019) reported that the purified ORP1 ORD protein transports cholesterol or DHE between liposomes *in vitro*, and its transfer activity is enhanced in the presence of PI(3,4)P_2_ or PI(4,5)P_2_ in the acceptor liposomes. However, the backward transfer of those phosphoinositides was not detected. Another study by Zhao and Ridgway (2017) reported that extraction of cholesterol from liposomes by purified full-length ORP1 protein was inhibited by the addition of PI4P, but not other phosphoinositides including PI(4,5)P_2_, to the liposomes. Consistent with this result, ORP1 protein extracts isotope-labeled PI4P from liposomes. Thus, these *in vitro* studies suggest that the ORP1 ORD is able to transport cholesterol, but may not transport phosphoinositides. In the cellular extent, ORP1L has been shown to mediate transport of cholesterol or PI4P (see below) at late endosome–ER MCSs. In the absence of exogenous low-density lipoprotein (LDL), which can be a source of late endosomal cholesterol *via* endocytosis, ORP1L mediates cholesterol transport to late endosomes from the ER, the site of its synthesis (Eden et al., 2016). This cholesterol transport can be driven by countertransport of PI4P in theory, but such countertransport has not yet been reported. Other studies also support the cholesterol transport, but its direction is opposite. Zhao and Ridgway (2017) demonstrated that accumulation of cholesterol in late endosomes in ORP1L-deficient HeLa cells was rescued by expression of wild-type, but not of mutants lacking the ORD or FFAT motif, suggesting that ORP1L mediates transfer of LDL-derived cholesterol from late endosomes to the ER along its concentration gradient. This ORP1L-mediated cholesterol transfer requires NPC1, which delivers LDL-cholesterol to the limiting membranes of late endosomes. Surprisingly, the mutant ORP1L, which is unable to bind PI4P due to disruption of the conserved PI4P-binding histidine residues in its ORD, did not rescue the cholesterol accumulation phenotype. This implies possible involvement of PI4P in the cholesterol transport, although it is difficult to reconcile at this moment how PI4P contributes to this cholesterol regulation. Dong et al. (2019) also supported the idea that ORP1L mediates cholesterol transport from late endosomes to the ER. Consistent with their *in vitro* data showing that the cholesterol transport activity of ORP1L is enhanced by PI(3,4)P_2_ or PI(4,5)P_2_, inhibition of PI(3,4)P_2_ synthesis by PI3KC2β on late endosomes (Marat et al., 2017) phenocopies the cholesterol transport defect. Collectively, ORP1L functions as a cholesterol transporter with or without the help of phosphoinositides, but its transport might be bi-directional between the ER and late endosomes/lysosomes depending on the cholesterol concentration.

#### Phosphatidylinositol 4-Phosphate Transport by ORP1

PI4P transport by ORP1L has been demonstrated at the MCSs between the ER and phagosomes. Phagosomes are endocytic organelles that engulf extracellular materials including microorganisms and apoptotic cells, and eventually fuse with lysosomes that degrade them. It has recently been demonstrated that ORP1L localizes at the MCSs between the ER and phagolysosomes, the mature phagosomes, where it mediates transport of PI4P from phagolysosomes to the ER (Levin-Konigsberg et al., 2019). The disappearance of PI4P from phagolysosomes, evaluated by live imaging, was delayed in ORP1L KO RAW 264.7 cells, and this delay was rescued by reexpression of wild-type ORP1L but not of the PI4P-binding mutant or the FFAT motif mutant. This ORP1L-mediated PI4P transport contributes to the segregation and concentration of this lipid into a domain that recruits the SKIP–ARL8B–kinesin complex (*via* PI4P binding of SKIP PH domain) leading to tubulation and fission of PI4P-positive membranes of phagolysosomes. Given that membrane-associated free cholesterol increases with phagosome maturation (Rai et al., 2016), such ORP1L-mediated PI4P transport to the ER might be coupled to back-transfer of cholesterol to the phagolysosomes. This interesting possibility needs further investigation.

#### Cholesterol Sensing by ORP1

ORP1L has been shown to control dynamics of late endosomes/lysosomes as a cholesterol sensor. Rab7 is a small GTPase that localizes at those organelles and controls a variety of their functions including subcellular positioning (Zerial and McBride, 2001; Cabukusta and Neefjes, 2018). ORP1L localizes at late endosomes/lysosomes *via* its PH domain and binding to Rab7 as an effector. Rab7 also recruits another effector RILP (Cantalupo et al., 2001; Jordens et al., 2001), which is the adaptor protein connecting Rab7 to the dynein–dynactin motor complex by binding to the light intermediate chain of dynein (Schroeder et al., 2014) and p150^Glued^ subunit of dynactin (Johansson et al., 2007). Interaction of Rab7–ORP1L–RILP to the dynein–dynactin motor complex, thus, determines the positioning of late endosomes/lysosomes, but this interaction is controlled by ORP1L-mediated cholesterol sensing. When the cholesterol levels are high in the limiting membrane of late endosomes, ORP1L accommodates cholesterol, leading to a conformation that does not allow it to bind VAP and, thus, to form MCSs with the ER. This, in turn, allows RILP to interact with the dynein–dynactin complex, and then late endosomes/lysosomes are clustered at the perinuclear area due to minus end-directed movement on microtubules. In a condition where cholesterol levels are low in late endosomes/lysosomes, ORP1L does not bind cholesterol in its ORD, leading to a conformational change in ORP1L so that it binds to VAP through the FFAT motif and forms MCSs with the ER. Then RILP no longer binds the dynein–dynactin complex, thereby leading to more scattered peripheral localization of late endosomes (Rocha et al., 2009). Thus, ORP1L controls late endosome/lysosome positioning depending on cholesterol levels *via* connecting or disconnecting those organelles to microtubules *via* promoting or inhibiting the binding capacity of RILP to the dynein–dynactin motor complex. Similar regulation was also reported for autophagosomes (Wijdeven et al., 2016). How lipid transport or countertransport activity of ORP1L contributes to such positioning control of late endosomes/lysosomes is still unclear.

#### ORP3

ORP3 has been reported to localize at the MCSs between late endosomes and the nuclear envelope (NE), whose outer membrane is continuous with the ER membrane. Extracellular vesicles such as exosomes or microvesicles are taken up *via* endocytosis by recipient cells, and their contents (e.g., nucleic acids, proteins, or lipids) are then delivered into the cytosol or other compartments (Raposo and Stoorvogel, 2013; van Niel et al., 2018). However, the underlying mechanism of the delivery of EV components is not completely understood (Mathieu et al., 2019). Rappa et al. (2017) demonstrated that EV components are transported along the endocytic pathway to a subset of Rab7-positive late endosomes, which are located in the nucleoplasmic reticulum in a deep nuclear envelope invagination. A subsequent study by the same group showed that such late endosomes contact the outer nuclear membranes in nuclear envelope invagination through tethering by ORP3 and VAPA. Functional ablation of ORP3 or VAPA (but not VAPB) leads to malformation of NE-late endosome MCSs in the nucleoplasmic reticulum and inhibits the transport of EV components such as CD9 or nucleic acids into the nucleoplasm, suggesting that ORP3-mediated MCSs contribute to delivering EV contents to the nucleus (Santos et al., 2018). Given that the nucleoplasmic reticulum is involved in Ca^2+^ regulation (Echevarría et al., 2003), the localization and function of ORP3 at the NE-late endosome MCSs may also be coupled to Ca^2+^ regulation, as shown at the ER–PM MCSs (Weber-Boyvat et al., 2015a; D’Souza et al., 2020; Gulyás et al., 2020). However, the targeting mechanism and lipid transport function of ORP3 in moving EV components into the nucleus remains unknown.

### Endoplasmic Reticulum–Lipid Droplet Membrane Contact Sites

#### ORP2

ORP2 is a member of group II (Figure 2). It has an FFAT motif and ORD, but lacks a PH domain. The ORP2 ORD binds oxysterol, cholesterol, and phosphoinositides such as PI4P, PI(4,5)P_2_, PI(3,5)P_2_, and PI(3,4,5)P_3_ *in vitro* (Xu et al., 2001; Hynynen et al., 2005, 2009; Suchanek et al., 2007). ORP2 has been suggested to function at the lipid droplets (LDs). ORP2 localizes to the surface of LDs or MCSs between the ER and LDs. Loss of function studies suggest that ORP2 may control triacylglycerol metabolism as well as lipolysis in LDs (Weber-Boyvat et al., 2015b). How ORP2 targets the LDs, however, is unclear. A recent study showed the association of ORP2 with the COPI machinery, which has been demonstrated to transport proteins to the LDs (Soni et al., 2009; Wilfling et al., 2014). ORP2 might utilize COPI to target LDs. Whether and how ORP2 exerts its function as a lipid transporter/exchanger is also unknown.

#### ORP5

ORP5, but not ORP8, is shown to localize and function at the ER–LD MCSs. ORP5 localizes LDs upon oleate loading, and its localization is mediated by ORD. Mutations in the lipid binding residues of ORP5 ORD abolished its localization, suggesting that PI4P/PS transport activity is required for the LD targeting. ORP5 knockdown increased the size of LDs, although no morphological change was reported in the previous study by the same group (Du et al., 2011). ORP5 knockdown also leads to an increase in PI4P and a decrease in PS on the LD surface. PI4K2α, but not other PI4Ks, was responsible for the generation of a pool of PI4P on LDs in ORP5 KD cells. ORP5 has been proposed to control the function of LDs *via* PI4P/PS countertransport, which is similar to that at the ER–PM MCSs but is supported by a different kinase, PI4K2α. However, direct evidence of the PI4P/PS exchange at the ER–LD MCSs *in situ* seems to be rather weak. It is quite interesting to find that ORP5 has a pleiotropic function as a PI4P/PS exchanger at multiple MCSs. However, many questions remain elusive. What is the physiological significance of PS transport to LDs? How does PI4K2α, which is a palmitoylated endosomal protein (Balla et al., 2002; Balla and Balla, 2006), contribute to the synthesis of the pool of PI4P on LDs? How is the localization (and hence the MCS formation) of ORP5, which does not require its PH domain, precisely controlled? Answering those questions may advance our understanding of ORP5 functions as well as novel aspects of LD biology.

### Endoplasmic Reticulum–Mitochondria Membrane Contact Sites

#### ORP5/8

ORP5 as well as ORP8 seem to have pleiotropic functions at multiple MCSs. ORP5 and ORP8 have been shown to localize at the ER–mitochondria MCSs (Galmes et al., 2016). Immunofluorescence staining and immunogold electron microscopy revealed the localization of both ORP5 and ORP8 at the MCSs between the ER and mitochondria in HeLa cells. Fractionation experiments showing the enrichment of ORP5 and ORP8 in mitochondria−associated ER membranes (MAMs) also support their localization at the ER–mitochondria MCSs. Localization of ORP5/8 to the ER–mitochondria MCSs does not require their PH domain but, instead, requires their novel binding partner, protein tyrosine phosphatase-interacting protein-51 (PTPIP51). PTPIP51 is the mitochondrial outer membrane protein that localized at the ER–mitochondria MCSs *via* interaction with VAPB and is involved in Ca^2+^ regulation at the mitochondria (Stoica et al., 2014). The ORD of ORP5 and ORP8 interacts with PTPIP51, and this interaction is required for their localization to the MCSs. Interestingly, though, the ORD mutant that abolishes PS binding cannot localize at the MCSs due to loss of binding to PTPIP51. Given that PS is transported to mitochondria to be converted to PE, ORP5/8 may contribute to PS transport to this organelle for PE synthesis. However, whether ORP5 and ORP8 mediate countertransport of PI4P and PS has not been confirmed. Nevertheless, the presence of PI, the precursor of PI4P, in the mitochondrial membrane was reported (Pemberton et al., 2020b; Zewe et al., 2020). Furthermore, the presence of PI4P-containing vesicles at the ER–mitochondria MCSs was also recently reported, although this pool of PI4P was provided via vesicular transport from the TGN after synthesis by PI4K3β (Nagashima et al., 2020). These observations suggest an interesting possibility of a direct involvement of PI4P in PS transport to mitochondria mediated by ORP5 and ORP8. These important aspects of whether and how ORP5/8 and PI4P contribute to such a process need further investigations.

## Discussion

In eukaryotes, more than 1,000 species of lipid molecules are coordinated to support fundamental cellular activities. In order to be fully functional, each lipid must be correctly positioned at the right place at the right time. Non-vesicular lipid transport by LTPs, including ORPs, controls such spatiotemporal positioning of lipids in cells (Holthuis and Menon, 2014). LTPs transfer their own set of lipid ligands between cellular membranes. ORPs, originally identified as oxysterol-binding proteins, have now been characterized as sensors or transporters of multiple lipids at MCSs. Biochemical studies as well as structural analysis have demonstrated that ORPs have a multiple-ligand repertoire including not only cholesterol but also phospholipids such as phosphoinositides, PS, and/or PC. Moreover, ORPs widely localized at multiple MCSs in cells to mediate transport of their own different lipid ligands, suggesting a functional diversity of ORPs to handle numerous cellular processes.

A unique functional property of ORPs is the lipid countertransport function at MCSs. Several, but not all, ORPs have been shown to exchange two different lipids: PI4P as a common driver-ligand and another lipid as a cargo-ligand. In the case of ORP5/8, they form ER–PM MCSs where PI4P and PI(4,5)P_2_ are enriched, and transport PI4P along its concentration gradient to the ER where PI4P is dephosphorylated by the PI4P phosphatase Sac1 (Chung et al., 2015b; Ghai et al., 2017; Sohn et al., 2018). This PM-to-ER flow of PI4P ensures the ER-to-PM counter-directional transport of the cargo-ligand PS against its concentration gradient. PI4K3α and Sac1 generate a concentration gradient of PI4P, the driver of this lipid countertransport, while ORP5/8 are the operators of lipid exchange at MCSs.

Although a better understanding of the role of ORPs as lipid transporters/exchangers at MCSs is emerging, many questions arise and remain unanswered. First, we do not know whether all ORPs act as lipid exchangers. As described above, some ORPs function as lipid exchangers in a PI4P-dependent manner. Considering that PI4P is distributed at cellular membranes such as the PM, Golgi, endosomes, and lysosomes (Hammond et al., 2014) (Figure 1), PI4P-driven lipid countertransport would be widely operated by ORPs at MCSs between those PI4P-containing membranes and the ER. In fact, structural analysis has suggested that PI4P might be the common ligand of ORPs (Tong et al., 2013; Antonny et al., 2018). Nevertheless, some ORPs have shown no lipid exchange activity and behave as transporters or sensors of lipids, suggesting that all ORPs may not necessarily be an exchanger. Second, the dynamic nature of ORPs at MCSs need to be understood. From a metabolic standpoint, cellular lipids must be under tight control in their quantity, quality, and distribution in response to changes in cellular status. How do ORPs dynamically change their localization at MCSs? How is their lipid transport/countertransport activity regulated? Such questions, especially in the context of cellular lipid homeostasis, would be important issues. Third, technical and methodological advancement will greatly help our understanding of the role of ORPs. Detection or analysis of lipids at organellar levels by imaging techniques will continue to provide useful information. Manipulation of lipids as well as ORP proteins in cells by chemical biology or some other genetic techniques will also give us novel insights. Last but not the least, the physiological significance of ORP-mediated lipid transport/countertransport in the regulation of lipid metabolism as well as some other processes must be further investigated. In particular, how such lipid countertransport at MCSs directly regulates specific cellular functions other than lipid metabolism remains elusive. In addition, the physiological role of ORPs at tissue or animal levels is largely unknown. The physiological importance of the intracellular lipid transport/countertransport by LTPs is underscored by human diseases caused by defects in such regulation. As the connection between malfunctions of intracellular lipid transport and various human diseases becomes progressively evident, a comprehensive understanding of the role of intracellular lipid transport is increasingly important.

## Author Contributions

FN and AK wrote the manuscript and prepared the figures. Both authors contributed to the article and approved the submitted version.

## Conflict of Interest

The authors declare that the research was conducted in the absence of any commercial or financial relationships that could be construed as a potential conflict of interest.

## References

[B1] Albanesi J. Wang H. Sun H. Q. Levine B. Yin H. (2015). GABARAP-mediated targeting of PI4K2A/PI4KIIα to autophagosomes regulates PtdIns4P-dependent autophagosome-lysosome fusion. *Autophagy* 11 2127–2129. 10.1080/15548627.2015.1093718 26391226 PMC4824573

[B2] Antonietta, De Matteis M. Di Campli A. Godi A. (2005). The role of the phosphoinositides at the Golgi complex. *Biochim. Biophys. Acta Mol. Cell Res.* 1744 396–405. 10.1016/j.bbamcr.2005.04.013 15979509

[B3] Antonny B. Bigay J. Mesmin B. (2018). The oxysterol-binding protein cycle: burning off PI(4)P to transport cholesterol. *Annu. Rev. Biochem.* 87 809–837. 10.1146/annurev-biochem-061516-044924 29596003

[B4] Balla A. Balla T. (2006). Phosphatidylinositol 4-kinases: old enzymes with emerging functions. *Trends Cell Biol.* 16 351–361. 10.1016/j.tcb.2006.05.003 16793271

[B5] Balla A. Tuymetova G. Barshishat M. Geiszt M. Balla T. (2002). Characterization of type II phosphatidylinositol 4-kinase isoforms reveals association of the enzymes with endosomal vesicular compartments. *J. Biol. Chem.* 277 20041–20050. 10.1074/jbc.M111807200 11923287

[B6] Balla T. (2013). Phosphoinositides: tiny lipids with giant impact on cell regulation. *Physiol. Rev.* 93 1019–1137. 10.1152/physrev.00028.2012 23899561 PMC3962547

[B7] Balla T. Kim Y. J. Alvarez-Prats A. Pemberton J. (2019). Lipid dynamics at contact sites between the endoplasmic reticulum and other organelles. *Annu. Rev. Cell Dev. Biol.* 35 85–109. 10.1146/annurev-cellbio-100818-125251 31590585

[B8] Balla T. Sengupta N. Kim Y. J. (2020). Lipid synthesis and transport are coupled to regulate membrane lipid dynamics in the endoplasmic reticulum. *Biochim. Biophys. Acta Mol. Cell Biol. Lipids* 1865:158461. 10.1016/j.bbalip.2019.05.005 31108203 PMC6858525

[B9] Barylko B. Mao Y. S. Wlodarski P. Jung G. Binns D. D. Sun H. Q. (2009). Palmitoylation controls the catalytic activity and subcellular distribution of phosphatidylinositol 4-kinase IIα. *J. Biol. Chem.* 284 9994–10003. 10.1074/jbc.M900724200 19211550 PMC2665123

[B10] Baskin J. M. Wu X. Christiano R. Oh M. S. Schauder C. M. Gazzerro E. (2015). The leukodystrophy protein FAM126A (hyccin) regulates PtdIns(4)P synthesis at the plasma membrane. *Nat. Cell Biol.* 18 132–138. 10.1038/ncb3271 26571211 PMC4689616

[B11] Blagoveshchenskaya A. Cheong F. Y. Rohde H. M. Glover G. Knödler A. Nicolson T. (2008). Integration of Golgi trafficking and growth factor signaling by the lipid phosphatase SAC1. *J. Cell Biol.* 180 803–812. 10.1083/jcb.200708109 18299350 PMC2265582

[B12] Bohnert M. (2020). Tether me, tether me not-dynamic organelle contact sites in metabolic rewiring. *Dev. Cell* 54 212–225. 10.1016/j.devcel.2020.06.026 32693056

[B13] Bojjireddy N. Guzman-Hernandez M. L. Reinhard N. R. Jović M. Balla T. (2015). EFR3s are palmitoylated plasma membrane proteins that control responsiveness to G-protein-coupled receptors. *J. Cell. Sci.* 128 118–128. 10.1242/jcs.157495 25380825 PMC4282049

[B14] Boura E. Nencka R. (2015). Phosphatidylinositol 4-kinases_ function, structure, and inhibition. *Exp. Cell Res.* 337 136–145. 10.1016/j.yexcr.2015.03.028 26183104

[B15] Burgett A. W. G. Poulsen T. B. Wangkanont K. Anderson D. R. Kikuchi C. Shimada K. (2011). Natural products reveal cancer cell dependence on oxysterol-binding proteins. *Nat. Chem. Biol.* 7 639–647. 10.1038/nchembio.625 21822274 PMC3158287

[B16] Cabukusta B. Neefjes J. (2018). Mechanisms of lysosomal positioning and movement. *Traffic* 19 761–769. 10.1111/tra.12587 29900632 PMC6175085

[B17] Cantalupo G. Alifano P. Roberti V. Bruni C. B. Bucci C. (2001). Rab-interacting lysosomal protein (RILP): the Rab7 effector required for transport to lysosomes. *EMBO J.* 20 683–693. 10.1093/emboj/20.4.683 11179213 PMC145419

[B18] Cao M. Wu Y. Ashrafi G. McCartney A. J. Wheeler H. Bushong E. A. (2017). Parkinson sac domain mutation in synaptojanin 1 impairs clathrin uncoating at synapses and triggers dystrophic changes in dopaminergic axons. *Neuron* 93 882.e5–896.e5. 10.1016/j.neuron.2017.01.019 28231468 PMC5340420

[B19] Castellano B. M. Thelen A. M. Moldavski O. Feltes M. van der Welle R. E. N. Mydock-McGrane L. (2017). Lysosomal cholesterol activates mTORC1 via an SLC38A9-Niemann-Pick C1 signaling complex. *Science* 355 1306–1311. 10.1126/science.aag1417 28336668 PMC5823611

[B20] Charman M. Colbourne T. R. Pietrangelo A. Kreplak L. Ridgway N. D. (2014). Oxysterol-binding protein (OSBP)-related protein 4 (ORP4) is essential for cell proliferation and survival. *J. Biol. Chem.* 289 15705–15717. 10.1074/jbc.M114.571216 24742681 PMC4140924

[B21] Chen M. Wen T. Horn H. T. Chandrahas V. K. Thapa N. Choi S. (2020). The nuclear phosphoinositide response to stress. *Cell Cycle* 19 268–289. 10.1080/15384101.2019.1711316 31902273 PMC7028212

[B22] Chung J. Nakatsu F. Baskin J. M. De Camilli P. (2015a). Plasticity of PI4KIIIα interactions at the plasma membrane. *EMBO Rep.* 16 312–320. 10.15252/embr.201439151 25608530 PMC4364870

[B23] Chung J. Torta F. Masai K. Lucast L. Czapla H. Tanner L. B. (2015b). Intracellular transport. *PI*4P/phosphatidylserine countertransport at ORP5- and ORP8-mediated ER-plasma membrane contacts. *Science* 349 428–432. 10.1126/science.aab1370 26206935 PMC4638224

[B24] de Saint-Jean M. Delfosse V. Douguet D. Chicanne G. Payrastre B. Bourguet W. (2011). Osh4p exchanges sterols for phosphatidylinositol 4-phosphate between lipid bilayers. *J. Cell Biol.* 195 965–978. 10.1083/jcb.201104062 22162133 PMC3241724

[B25] De Tito S. Hervás J. H. van Vliet A. R. Tooze S. A. (2020). The golgi as an assembly line to the autophagosome. *Trends Biochem. Sci.* 45 484–496. 10.1016/j.tibs.2020.03.010 32307224

[B26] Del Bel L. M. Brill J. A. (2018). Sac1, a lipid phosphatase at the interface of vesicular and nonvesicular transport. *Traffic* 19 301–318. 10.1111/tra.12554 29411923

[B27] Di Paolo G. De Camilli P. (2006). Phosphoinositides in cell regulation and membrane dynamics. *Nature* 443 651–657. 10.1038/nature05185 17035995

[B28] Dickson E. J. Jensen J. B. Vivas O. Kruse M. Traynor-Kaplan A. E. Hille B. (2016). Dynamic formation of ER-PM junctions presents a lipid phosphatase to regulate phosphoinositides. *J. Cell Biol.* 213 33–48. 10.1083/jcb.201508106 27044890 PMC4828688

[B29] Dong J. Du X. Wang H. Wang J. Lu C. Chen X. (2019). Allosteric enhancement of ORP1-mediated cholesterol transport by PI(4,5)P2/PI(3,4)P2. *Nat. Commun.* 10 829–816. 10.1038/s41467-019-08791-0 30783101 PMC6381110

[B30] Dong R. Saheki Y. Swarup S. Lucast L. Harper J. W. De Camilli P. (2016). Endosome-ER contacts control actin nucleation and retromer function through VAP-dependent regulation of PI4P. *Cell* 166 408–423. 10.1016/j.cell.2016.06.037 27419871 PMC4963242

[B31] Dong R. Zhu T. Benedetti L. Gowrishankar S. Deng H. Cai Y. (2018). The inositol 5-phosphatase INPP5K participates in the fine control of ER organization. *J. Cell Biol.* 217 3577–3592. 10.1083/jcb.201802125 30087126 PMC6168264

[B32] D’Souza R. S. Lim J. Y. Turgut A. Servage K. Zhang J. Orth K. (2020). Calcium-stimulated disassembly of focal adhesions mediated by an ORP3/IQSec1 complex. *eLife* 9:1381. 10.7554/eLife.54113 32234213 PMC7159923

[B33] Du X. Kumar J. Ferguson C. Schulz T. A. Ong Y. S. Hong W. (2011). A role for oxysterol-binding protein-related protein 5 in endosomal cholesterol trafficking. *J. Cell Biol.* 192 121–135. 10.1083/jcb.201004142 21220512 PMC3019559

[B34] Echevarría W. Leite M. F. Guerra M. T. Zipfel W. R. Nathanson M. H. (2003). Regulation of calcium signals in the nucleus by a nucleoplasmic reticulum. *Nat. Cell Biol.* 5 440–446. 10.1038/ncb980 12717445 PMC3572851

[B35] Eden E. R. Sanchez-Heras E. Tsapara A. Sobota A. Levine T. P. Futter C. E. (2016). Annexin A1 tethers membrane contact sites that mediate ER to endosome cholesterol transport. *Dev. Cell* 37 473–483. 10.1016/j.devcel.2016.05.005 27270042 PMC4906250

[B36] Eisenberg-Bord M. Shai N. Schuldiner M. Bohnert M. (2016). A tether is a tether is a tether: tethering at membrane contact sites. *Dev. Cell* 39 395–409. 10.1016/j.devcel.2016.10.022 27875684

[B37] Fournier M. V. Guimarães, da Costa F. Paschoal M. E. Ronco L. V. Carvalho M. G. (1999). Identification of a gene encoding a human oxysterol-binding protein-homologue: a potential general molecular marker for blood dissemination of solid tumors. *Cancer Res.* 59 3748–3753. 10446991

[B38] Galmes R. Houcine A. van Vliet A. R. Agostinis P. Jackson C. L. Giordano F. (2016). ORP5/ORP8 localize to endoplasmic reticulum-mitochondria contacts and are involved in mitochondrial function. *EMBO Rep.* 17 800–810. 10.15252/embr.201541108 27113756 PMC5278607

[B39] Ghai R. Du X. Wang H. Dong J. Ferguson C. Brown A. J. (2017). ORP5 and ORP8 bind phosphatidylinositol-4, 5-biphosphate (PtdIns(4,5)P 2) and regulate its level at the plasma membrane. *Nat. Commun.* 8:757. 10.1038/s41467-017-00861-5 28970484 PMC5624964

[B40] Godi A. Pertile P. Meyers R. Marra P. Di Tullio G. Iurisci C. (1999). ARF mediates recruitment of PtdIns-4-OH kinase-beta and stimulates synthesis of PtdIns(4,5)P2 on the Golgi complex. *Nat. Cell Biol.* 1 280–287. 10.1038/12993 10559940

[B41] Goto A. Charman M. Ridgway N. D. (2016). Oxysterol-binding protein activation at endoplasmic reticulum-golgi contact sites reorganizes phosphatidylinositol 4-phosphate pools. *J. Biol. Chem.* 291 1336–1347. 10.1074/jbc.M115.682997 26601944 PMC4714219

[B42] Gulyás G. Sohn M. Kim Y. J. Varnai P. Balla T. (2020). ORP3 phosphorylation regulates phosphatidylinositol 4-phosphate and Ca2+ dynamics at plasma membrane-ER contact sites. *J. Cell. Sci.* 133:jcs237388. 10.1242/jcs.237388 32041906 PMC7097422

[B43] Guo S. Stolz L. E. Lemrow S. M. York J. D. (1999). SAC1-like domains of yeast SAC1, INP52, and INP53 and of human synaptojanin encode polyphosphoinositide phosphatases. *J. Biol. Chem.* 274 12990–12995. 10.1074/jbc.274.19.12990 10224048

[B44] Gurung R. Tan A. Ooms L. M. McGrath M. J. Huysmans R. D. Munday A. D. (2003). Identification of a novel domain in two mammalian inositol-polyphosphate 5-phosphatases that mediates membrane ruffle localization. The inositol 5-phosphatase skip localizes to the endoplasmic reticulum and translocates to membrane ruffles following epidermal growth factor stimulation. *J. Biol. Chem.* 278 11376–11385. 10.1074/jbc.M209991200 12536145

[B45] Hammond G. R. V. Machner M. P. Balla T. (2014). A novel probe for phosphatidylinositol 4-phosphate reveals multiple pools beyond the Golgi. *J. Cell Biol.* 205 113–126. 10.1083/jcb.201312072 24711504 PMC3987136

[B46] Hausser A. Storz P. Märtens S. Link G. Toker A. Pfizenmaier K. (2005). Protein kinase D regulates vesicular transport by phosphorylating and activating phosphatidylinositol-4 kinase IIIβ at the Golgi complex. *Nat. Cell Biol.* 7 880–886. 10.1038/ncb1289 16100512 PMC1458033

[B47] Helle S. C. J. Kanfer G. Kolar K. Lang A. Michel A. H. Kornmann B. (2013). Organization and function of membrane contact sites. *Biochim. Biophys. Acta* 1833 2526–2541. 10.1016/j.bbamcr.2013.01.028 23380708

[B48] Henriques Silva N. Vasconcellos Fournier M. Pimenta G. Pulcheri W. A. Spector N. Costa da (2003). HLM/OSBP2 is expressed in chronic myeloid leukemia. *Int. J. Mol. Med.* 12 663–666. 12964051

[B49] Holthuis J. C. M. Levine T. P. (2005). Lipid traffic: floppy drives and a superhighway. *Nat. Rev. Mol. Cell Biol.* 6 209–220. 10.1038/nrm1591 15738987

[B50] Holthuis J. C. M. Menon A. K. (2014). Lipid landscapes and pipelines in membrane homeostasis. *Nature* 510 48–57. 10.1038/nature13474 24899304

[B51] Hsu F. Mao Y. (2013). The Sac domain-containing phosphoinositide phosphatases: structure, function, and disease. *Front. Biol.* 8:395–407. 10.1007/s11515-013-1258-y 24860601 PMC4031025

[B52] Hsu F. Hu F. Mao Y. (2015). Spatiotemporal control of phosphatidylinositol 4-phosphate by Sac2 regulates endocytic recycling. *J. Cell Biol.* 209 97–110. 10.1083/jcb.201408027 25869669 PMC4395482

[B53] Hung C.-S. Lin Y.-L. Wu C.-I. Huang C.-J. Ting L.-P. (2009). Suppression of hepatitis B viral gene expression by phosphoinositide 5-phosphatase SKIP. *Cell. Microbiol.* 11 37–50. 10.1111/j.1462-5822.2008.01235.x 18774950

[B54] Hynynen R. Laitinen S. Käkelä R. Tanhuanpää K. Lusa S. Ehnholm C. (2005). Overexpression of OSBP-related protein 2 (ORP2) induces changes in cellular cholesterol metabolism and enhances endocytosis. *Biochem. J.* 390 273–283. 10.1042/BJ20042082 15859942 PMC1184581

[B55] Hynynen R. Suchanek M. Spandl J. Bäck N. Thiele C. Olkkonen V. M. (2009). OSBP-related protein 2 is a sterol receptor on lipid droplets that regulates the metabolism of neutral lipids. *J. Lipid Res.* 50 1305–1315. 10.1194/jlr.M800661-JLR200 19224871 PMC2694330

[B56] Ijuin T. Takenawa T. (2003). SKIP negatively regulates insulin-induced GLUT4 translocation and membrane ruffle formation. *Mol. Cell Biol.* 23 1209–1220. 10.1128/mcb.23.4.1209-1220.2003 12556481 PMC141139

[B57] Im Y. J. Raychaudhuri S. Prinz W. A. Hurley J. H. (2005). Structural mechanism for sterol sensing and transport by OSBP-related proteins. *Nature* 437 154–158. 10.1038/nature03923 16136145 PMC1431608

[B58] Johansson M. Lehto M. Tanhuanpää K. Cover T. L. Olkkonen V. M. (2005). The oxysterol-binding protein homologue ORP1L interacts with Rab7 and alters functional properties of late endocytic compartments. *Mol. Biol. Cell* 16 5480–5492. 10.1091/mbc.e05-03-0189 16176980 PMC1289395

[B59] Johansson M. Rocha N. Zwart W. Jordens I. Janssen L. Kuijl C. (2007). Activation of endosomal dynein motors by stepwise assembly of Rab7-RILP-p150Glued, ORP1L, and the receptor betalll spectrin. *J. Cell Biol.* 176 459–471. 10.1083/jcb.200606077 17283181 PMC2063981

[B60] Jordens I. Fernandez-Borja M. Marsman M. Dusseljee S. Janssen L. Calafat J. (2001). The Rab7 effector protein RILP controls lysosomal transport by inducing the recruitment of dynein-dynactin motors. *Curr. Biol.* 11 1680–1685. 10.1016/s0960-9822(01)00531-0 11696325

[B61] Judith D. Jefferies H. B. J. Boeing S. Frith D. Snijders A. P. Tooze S. A. (2019). ATG9A shapes the forming autophagosome through Arfaptin 2 and phosphatidylinositol 4-kinase IIIβ. *J. Cell Biol.* 218 1634–1652. 10.1083/jcb.201901115 30917996 PMC6504893

[B62] Kuge O. Nishijima M. Akamatsu Y. (1991). A Chinese hamster cDNA encoding a protein essential for phosphatidylserine synthase I activity. *J. Biol. Chem.* 266 24184–24189. 10.1016/s0021-9258(18)54410-0 1748687

[B63] Kuge O. Saito K. Nishijima M. (1997). Cloning of a Chinese hamster ovary (CHO) cDNA encoding phosphatidylserine synthase (PSS) II, overexpression of which suppresses the phosphatidylserine biosynthetic defect of a PSS I-lacking mutant of CHO-K1 cells. *J. Biol. Chem.* 272 19133–19139. 10.1074/jbc.272.31.19133 9235902

[B64] Lehto M. Hynynen R. Karjalainen K. Kuismanen E. Hyvärinen K. Olkkonen V. M. (2005). Targeting of OSBP-related protein 3 (ORP3) to endoplasmic reticulum and plasma membrane is controlled by multiple determinants. *Exp. Cell Res.* 310 445–462. 10.1016/j.yexcr.2005.08.003 16143324

[B65] Lehto M. Laitinen S. Chinetti G. Johansson M. Ehnholm C. Staels B. (2001). The OSBP-related protein family in humans. *J. Lipid Res.* 42 1203–1213. 10.1016/s0022-2275(20)31570-4 11483621

[B66] Lemmon M. A. (2008). Membrane recognition by phospholipid-binding domains. *Nat. Rev. Mol. Cell Biol.* 9 99–111. 10.1038/nrm2328 18216767

[B67] Lenz W. D. Majewski F. (1974). A generalized disorders of the connective tissues with progeria, choanal atresia, symphalangism, hypoplasia of dentine and craniodiaphyseal hypostosis. *Birth Defects Orig. Artic Ser.* 10 133–136. 4376705

[B68] Lev S. (2012). Nonvesicular lipid transfer from the endoplasmic reticulum. *Cold Spring Harb. Perspect. Biol.* 4:a013300. 10.1101/cshperspect.a013300 23028121 PMC3475164

[B69] Levin R. Hammond G. R. V. Balla T. De Camilli P. Fairn G. D. Grinstein S. (2017). Multiphasic dynamics of phosphatidylinositol 4-phosphate during phagocytosis. *Mol. Biol. Cell* 28 128–140. 10.1091/mbc.E16-06-0451 28035045 PMC5221617

[B70] Levine T. P. Munro S. (1998). The pleckstrin homology domain of oxysterol-binding protein recognises a determinant specific to Golgi membranes. *Curr. Biol.* 8 729–739. 10.1016/s0960-9822(98)70296-9 9651677

[B71] Levine T. P. Munro S. (2002). Targeting of Golgi-specific pleckstrin homology domains involves both PtdIns 4-kinase-dependent and -independent components. *Curr. Biol.* 12 695–704. 10.1016/s0960-9822(02)00779-0 12007412

[B72] Levin-Konigsberg R. Montaño-Rendón F. Keren-Kaplan T. Li R. Ego B. Mylvaganam S. (2019). Phagolysosome resolution requires contacts with the endoplasmic reticulum and phosphatidylinositol-4-phosphate signalling. *Nat. Cell Biol.* 21 1234–1247. 10.1038/s41556-019-0394-2 31570833 PMC8340083

[B73] Lim C.-Y. Davis O. B. Shin H. R. Zhang J. Berdan C. A. Jiang X. (2019). ER-lysosome contacts enable cholesterol sensing by mTORC1 and drive aberrant growth signalling in Niemann-Pick type C. *Nat. Cell Biol.* 21 1206–1218. 10.1038/s41556-019-0391-5 31548609 PMC6936960

[B74] Liu X. Ridgway N. D. (2014). Characterization of the sterol and phosphatidylinositol 4-phosphate binding properties of Golgi-associated OSBP-related protein 9 (ORP9). *PLoS One* 9:e108368. 10.1371/journal.pone.0108368 25255026 PMC4177916

[B75] Loewen C. J. R. Roy A. Levine T. P. (2003). A conserved ER targeting motif in three families of lipid binding proteins and in Opi1p binds VAP. *EMBO J.* 22 2025–2035. 10.1093/emboj/cdg201 12727870 PMC156073

[B76] Lu D. Sun H. Q. Wang H. Barylko B. Fukata Y. Fukata M. (2012). Phosphatidylinositol 4-Kinase IIÎ ± Is palmitoylated by golgi-localized palmitoyltransferases in cholesterol-dependent manner. *J. Biol. Chem.* 287 21856–21865. 10.1074/jbc.M112.348094 22535966 PMC3381148

[B77] Machaca K. (2020). Ca2+ signaling and lipid transfer “pas a deux” at ER-PM contact sites orchestrate cell migration. *Cell Calc.* 89:102226. 10.1016/j.ceca.2020.102226 32505782

[B78] Maeda K. Anand K. Chiapparino A. Kumar A. Poletto M. Kaksonen M. (2013). Interactome map uncovers phosphatidylserine transport by oxysterol-binding proteins. *Nature* 501 257–261. 10.1038/nature12430 23934110

[B79] Mani M. Lee S. Y. Lucast L. Cremona O. Di Paolo G. De Camilli P. (2007). The dual phosphatase activity of synaptojanin1 is required for both efficient synaptic vesicle endocytosis and reavailability at nerve terminals. *Neuron* 56 1004–1018. 10.1016/j.neuron.2007.10.032 18093523 PMC3653591

[B80] Marat A. L. Wallroth A. Lo W.-T. Müller R. Norata G. D. Falasca M. (2017). mTORC1 activity repression by late endosomal phosphatidylinositol 3,4-bisphosphate. *Science* 356 968–972. 10.1126/science.aaf8310 28572395

[B81] Mathieu M. Martin-Jaular L. Lavieu G. Théry C. (2019). Specificities of secretion and uptake of exosomes and other extracellular vesicles for cell-to-cell communication. *Nat. Cell Biol.* 21 9–17. 10.1038/s41556-018-0250-9 30602770

[B82] McGrath M. J. Eramo M. J. Gurung R. Sriratana A. Gehrig S. M. Lynch G. S. (2021). Defective lysosome reformation during autophagy causes skeletal muscle disease. *J. Clin. Invest.* 131:e135124. 10.1172/JCI135124 33119550 PMC7773396

[B83] McPherson P. S. Garcia E. P. Slepnev V. I. David C. Zhang X. Grabs D. (1996). A presynaptic inositol-5-phosphatase. *Nature* 379 353–357. 10.1038/379353a0 8552192

[B84] Mesmin B. Antonny B. (2016). The counterflow transport of sterols and PI4P. *Biochim. Biophys. Acta* 1861 940–951. 10.1016/j.bbalip.2016.02.024 26928592

[B85] Mesmin B. Bigay J. Moser, von Filseck J. Lacas-Gervais S. Drin G. (2013). A four-step cycle driven by PI(4)P hydrolysis directs sterol/PI(4)P exchange by the ER-Golgi tether OSBP. *Cell* 155 830–843. 10.1016/j.cell.2013.09.056 24209621

[B86] Mesmin B. Bigay J. Polidori J. Jamecna D. Lacas-Gervais S. Antonny B. (2017). Sterol transfer, PI4P consumption, and control of membrane lipid order by endogenous OSBP. *EMBO J.* 36 3156–3174. 10.15252/embj.201796687 28978670 PMC5666618

[B87] Mochizuki S. Miki H. Zhou R. Kido Y. Nishimura W. Kikuchi M. (2018). Oxysterol-binding protein-related protein (ORP) 6 localizes to the ER and ER-plasma MCS sites and is involved in the turnover of PI4P in cerebellar granule neurons. *Exp. Cell Res.* 370 601–612. 10.1016/j.yexcr.2018.07.025 30028970

[B88] Moser, von Filseck J. Čopič A. Delfosse V. Vanni S. Jackson C. L. (2015). Intracellular transport. Phosphatidylserine transport by ORP/Osh proteins is driven by phosphatidylinositol 4-phosphate. *Science* 349 432–436. 10.1126/science.aab1346 26206936

[B89] Murphy S. E. Levine T. P. (2016). VAP, a versatile access point for the endoplasmic reticulum: review and analysis of FFAT-like motifs in the VAPome. *Biochim. Biophys. Acta* 1861 952–961. 10.1016/j.bbalip.2016.02.009 26898182

[B90] Nagashima S. Tábara L.-C. Tilokani L. Paupe V. Anand H. Pogson J. H. (2020). Golgi-derived PI(4)P-containing vesicles drive late steps of mitochondrial division. *Science* 367 1366–1371. 10.1126/science.aax6089 32193326

[B91] Nakatsu F. Baskin J. M. Chung J. Tanner L. B. Shui G. Lee S. Y. (2012). PtdIns4P synthesis by PI4KIIIα at the plasma membrane and its impact on plasma membrane identity. *J. Cell Biol.* 199 1003–1016. 10.1083/jcb.201206095 23229899 PMC3518224

[B92] Nakatsu F. Messa M. Nández R. Czapla H. Zou Y. Strittmatter S. M. (2015). Sac2/INPP5F is an inositol 4-phosphatase that functions in the endocytic pathway. *J. Cell Biol.* 209 85–95. 10.1083/jcb.201409064 25869668 PMC4395491

[B93] Nemoto Y. Wenk M. R. Watanabe M. Daniell L. Murakami T. Ringstad N. (2001). Identification and characterization of a synaptojanin 2 splice isoform predominantly expressed in nerve terminals. *J. Biol. Chem.* 276 41133–41142. 10.1074/jbc.M106404200 11498538

[B94] Ngo M. Ridgway N. D. (2009). Oxysterol binding protein-related Protein 9 (ORP9) is a cholesterol transfer protein that regulates Golgi structure and function. *Mol. Biol. Cell* 20 1388–1399. 10.1091/mbc.e08-09-0905 19129476 PMC2649274

[B95] Nguyen P. M. Gandasi N. R. Xie B. Sugahara S. Xu Y. Idevall-Hagren O. (2019). The PI(4)P phosphatase Sac2 controls insulin granule docking and release. *J. Cell Biol.* 218 3714–3729. 10.1083/jcb.201903121 31533953 PMC6829663

[B96] Nishimura T. Gecht M. Covino R. Hummer G. Surma M. A. Klose C. (2019). Osh proteins control nanoscale lipid organization necessary for PI(4,5)P2 synthesis. *Mol. Cell* 75 1043.e8–1057.e8. 10.1016/j.molcel.2019.06.037 31402097 PMC6739424

[B97] Olkkonen V. M. (2015). OSBP-related protein family in lipid transport over membrane contact sites. *Lipid Insights* 8s1 1726–1729. 10.4137/LPI.S31726 26715851 PMC4685180

[B98] Pemberton J. G. Kim Y. J. Balla T. (2020a). Integrated regulation of the phosphatidylinositol cycle and phosphoinositide-driven lipid transport at ER-PM contact sites. *Traffic* 21 200–219. 10.1111/tra.12709 31650663

[B99] Pemberton J. G. Kim Y. J. Humpolickova J. Eisenreichova A. Sengupta N. Tóth D. J. (2020b). Defining the subcellular distribution and metabolic channeling of phosphatidylinositol. *J. Cell Biol.* 219 213–234. 10.1083/jcb.201906130 32211894 PMC7054996

[B100] Péresse T. Kovacs D. Subra M. Bigay J. Tsai M.-C. Polidori J. (2020). Molecular and cellular dissection of the oxysterol-binding protein cycle through a fluorescent inhibitor. *J. Biol. Chem.* 295 4277–4288. 10.1074/jbc.ra119.012012 32075908 PMC7105299

[B101] Phillips M. J. Voeltz G. K. (2015). Structure and function of ER membrane contact sites with other organelles. *Nat. Publ. Group* 17 69–82. 10.1038/nrm.2015.8 26627931 PMC5117888

[B102] Pietrangelo A. Ridgway N. D. (2018). Bridging the molecular and biological functions of the oxysterol-binding protein family. *Cell. Mol. Life Sci.* 75 3079–3098. 10.1007/s00018-018-2795-y 29536114 PMC11105248

[B103] Pirruccello M. De Camilli P. (2012). Inositol 5-phosphatases: insights from the Lowe syndrome protein OCRL. *Trends Biochem. Sci.* 37 134–143. 10.1016/j.tibs.2012.01.002 22381590 PMC3323734

[B104] Prinz W. A. Toulmay A. Balla T. (2019). The functional universe of membrane contact sites. *Nat. Rev. Mol. Cell Biol.* 0 1–18. 10.1038/s41580-019-0180-9 31732717 PMC10619483

[B105] Rai A. Pathak D. Thakur S. Singh S. Dubey A. K. Mallik R. (2016). Dynein clusters into lipid microdomains on phagosomes to drive rapid transport toward lysosomes. *Cell* 164 722–734. 10.1016/j.cell.2015.12.054 26853472 PMC4752818

[B106] Rameh L. E. Arvidsson A. K. Carraway K. L. Couvillon A. D. Rathbun G. Crompton A. (1997). A comparative analysis of the phosphoinositide binding specificity of pleckstrin homology domains. *J. Biol. Chem.* 272 22059–22066. 10.1074/jbc.272.35.22059 9268346

[B107] Ramos A. R. Ghosh S. Suhel T. Chevalier C. Obeng E. O. Fafilek B. (2020). Phosphoinositide 5-phosphatases SKIP and SHIP2 in ruffles, the endoplasmic reticulum and the nucleus: an update. *Adv. Biol. Regul.* 75:100660. 10.1016/j.jbior.2019.100660 31628071

[B108] Raposo G. Stoorvogel W. (2013). Extracellular vesicles: exosomes, microvesicles, and friends. *J. Cell Biol.* 200 373–383. 10.1083/jcb.201212113 23420871 PMC3575529

[B109] Rappa G. Santos M. F. Green T. M. Karbanová J. Hassler J. Bai Y. (2017). Nuclear transport of cancer extracellular vesicle-derived biomaterials through nuclear envelope invagination-associated late endosomes. *Oncotarget* 8 14443–14461. 10.18632/oncotarget.14804 28129640 PMC5362417

[B110] Raychaudhuri S. Prinz W. A. (2010). The diverse functions of oxysterol-binding proteins. *Annu. Rev. Cell Dev. Biol.* 26 157–177. 10.1146/annurev.cellbio.042308.113334 19575662 PMC3478074

[B111] Raychaudhuri S. Im Y. J. Hurley J. H. Prinz W. A. (2006). Nonvesicular sterol movement from plasma membrane to ER requires oxysterol-binding protein-related proteins and phosphoinositides. *J. Cell Biol.* 173 107–119. 10.1083/jcb.200510084 16585271 PMC2063795

[B112] Reinisch K. M. Prinz W. A. (2021). Mechanisms of nonvesicular lipid transport. *J. Cell Biol.* 220:e202012058. 10.1083/jcb.202012058 33605998 PMC7901144

[B113] Rocha N. Kuijl C. van der Kant R. Janssen L. Houben D. Janssen H. (2009). Cholesterol sensor ORP1L contacts the ER protein VAP to control Rab7-RILP-p150 Glued and late endosome positioning. *J. Cell Biol.* 185 1209–1225. 10.1083/jcb.200811005 19564404 PMC2712958

[B114] Santos A. L. Preta G. (2018). Lipids in the cell: organisation regulates function. *Cell. Mol. Life Sci.* 75 1909–1927. 10.1007/s00018-018-2765-4 29427074 PMC11105414

[B115] Santos M. F. Rappa G. Karbanová J. Kurth T. Corbeil D. Lorico A. (2018). VAMP-associated protein-A and oxysterol-binding protein-related protein 3 promote the entry of late endosomes into the nucleoplasmic reticulum. *J. Biol. Chem.* 293 13834–13848. 10.1074/jbc.RA118.003725 30018135 PMC6130944

[B116] Santos N. C. Girik V. Nunes-Hasler P. (2020). ORP5 and ORP8: sterol sensors and phospholipid transfer proteins at membrane contact sites? *Biomolecules* 10:928. 10.3390/biom10060928 32570981 PMC7356933

[B117] Sasaki J. Ishikawa K. Arita M. Taniguchi K. (2011). ACBD3-mediated recruitment of PI4KB to picornavirus RNA replication sites. *EMBO J.* 31 754–766. 10.1038/emboj.2011.429 22124328 PMC3273392

[B118] Schroeder C. M. Ostrem J. M. L. Hertz N. T. Vale R. D. (2014). A Ras-like domain in the light intermediate chain bridges the dynein motor to a cargo-binding region. *eLife* 3:e03351. 10.7554/eLife.03351 25272277 PMC4359372

[B119] Scorrano L. De Matteis M. A. Emr S. Giordano F. Hajnóczky G. Kornmann B. (2019). Coming together to define MCS sites. *Nat. Commun.* 10 1287–1211. 10.1038/s41467-019-09253-3 30894536 PMC6427007

[B120] Silva N. H. Pimenta G. Pulcheri W. A. Fournier M. V. Spector N. Costa da (2001). Detection of messenger RNA in leukocytes or plasma of patients with chronic myeloid leukemia. *Oncol. Rep.* 8 693–696. 10.3892/or.8.3.693 11295104

[B121] Sobajima T. Yoshimura S.-I. Maeda T. Miyata H. Miyoshi E. Harada A. (2018). The Rab11-binding protein RELCH/KIAA1468 controls intracellular cholesterol distribution. *J. Cell Biol.* 217 1777–1796. 10.1083/jcb.201709123 29514919 PMC5940305

[B122] Sohn M. Ivanova P. Brown H. A. Tóth D. J. Varnai P. Kim Y. J. (2016). Lenz-Majewski mutations in PTDSS1 affect phosphatidylinositol 4-phosphate metabolism at ER-PM and ER-Golgi junctions. *Proc. Natl. Acad. Sci. U.S.A.* 113 4314–4319. 10.1073/pnas.1525719113 27044099 PMC4843478

[B123] Sohn M. Korzeniowski M. Zewe J. P. Wills R. C. Hammond G. R. V. Humpolickova J. (2018). PI(4,5)P2 controls plasma membrane PI4P and PS levels via ORP5/8 recruitment to ER-PM contact sites. *J. Cell Biol.* 217 1797–1813. 10.1083/jcb.201710095 29472386 PMC5940310

[B124] Soni K. G. Mardones G. A. Sougrat R. Smirnova E. Jackson C. L. Bonifacino J. S. (2009). Coatomer-dependent protein delivery to lipid droplets. *J. Cell Sci.* 122 1834–1841. 10.1242/jcs.045849 19461073 PMC2684835

[B125] Sousa S. B. Jenkins D. Chanudet E. Tasseva G. Ishida M. Anderson G. (2014). Gain-of-function mutations in the phosphatidylserine synthase 1 (PTDSS1) gene cause Lenz-Majewski syndrome. *Nat. Genet.* 46 70–76. 10.1038/ng.2829 24241535

[B126] Stefan C. J. Manford A. G. Baird D. Yamada-Hanff J. Mao Y. Emr S. D. (2011). Osh proteins regulate phosphoinositide metabolism at ER-plasma membrane contact sites. *Cell* 144 389–401. 10.1016/j.cell.2010.12.034 21295699

[B127] Stefan C. J. Trimble W. S. Grinstein S. Drin G. Reinisch K. De Camilli P. (2017). Membrane dynamics and organelle biogenesis-lipid pipelines and vesicular carriers. *BMC Biol.* 15:102. 10.1186/s12915-017-0432-0 29089042 PMC5663033

[B128] Stoica R. De Vos K. J. Paillusson S. Mueller S. Sancho R. M. Lau K.-F. (2014). ER-mitochondria associations are regulated by the VAPB-PTPIP51 interaction and are disrupted by ALS/FTD-associated TDP-43. *Nat. Commun.* 5 3996–3912. 10.1038/ncomms4996 24893131 PMC4046113

[B129] Suchanek M. Hynynen R. Wohlfahrt G. Lehto M. Johansson M. Saarinen H. (2007). The mammalian oxysterol-binding protein-related proteins (ORPs) bind 25-hydroxycholesterol in an evolutionarily conserved pocket. *Biochem. J.* 405 473–480. 10.1042/BJ20070176 17428193 PMC2267293

[B130] Taylor F. R. Saucier S. E. Shown E. P. Parish E. J. Kandutsch A. A. (1984). Correlation between oxysterol binding to a cytosolic binding protein and potency in the repression of hydroxymethylglutaryl coenzyme A reductase. *J. Biol. Chem.* 259 12382–12387. 10.1016/s0021-9258(18)90757-x 6490619

[B131] Tong J. Yang H. Yang H. Eom S. H. Im Y. J. (2013). Structure of Osh3 reveals a conserved mode of phosphoinositide binding in oxysterol-binding proteins. *Structure* 21 1203–1213. 10.1016/j.str.2013.05.007 23791945

[B132] Udagawa O. Ito C. Ogonuki N. Sato H. Lee S. Tripvanuntakul P. (2014). Oligo-astheno-teratozoospermia in mice lacking ORP4, a sterol-binding protein in the OSBP-related protein family. *Genes Cells* 19 13–27. 10.1111/gtc.12105 24245814

[B133] van Meer G. de Kroon A. I. P. M. (2011). Lipid map of the mammalian cell. *J. Cell. Sci.* 124 5–8. 10.1242/jcs.071233 21172818

[B134] van Meer G. Voelker D. R. Feigenson G. W. (2008). Membrane lipids: where they are and how they behave. *Nat. Rev. Mol. Cell Biol.* 9 112–124. 10.1038/nrm2330 18216768 PMC2642958

[B135] van Niel G. D’Angelo G. Raposo G. (2018). Shedding light on the cell biology of extracellular vesicles. *Nat. Rev. Mol. Cell Biol.* 19 213–228. 10.1038/nrm.2017.125 29339798

[B136] Vance J. E. (2014). Phospholipid synthesis and transport in mammalian cells. *Traffic* 16 1–18. 10.1111/tra.12230 25243850

[B137] Venditti R. Masone M. C. Rega L. R. Di Tullio G. Santoro M. Polishchuk E. (2019a). The activity of Sac1 across ER–TGN contact sites requires the four-phosphate-adaptor-protein-1. *J. Cell Biol.* 218 783–797. 10.1083/jcb.201812021 30659099 PMC6400556

[B138] Venditti R. Rega L. R. Masone M. C. Santoro M. Polishchuk E. Sarnataro D. (2019b). Molecular determinants of ER-Golgi contacts identified through a new FRET-FLIM system. *J. Cell Biol.* 218 1055–1065. 10.1083/jcb.201812020 30659100 PMC6400564

[B139] Vihervaara T. Uronen R.-L. Wohlfahrt G. Björkhem I. Ikonen E. Olkkonen V. M. (2011). Sterol binding by OSBP-related protein 1L regulates late endosome motility and function. *Cell. Mol. Life Sci.* 68 537–551. 10.1007/s00018-010-0470-z 20690035 PMC11114714

[B140] Wang C. JeBailey L. Ridgway N. D. (2002). Oxysterol-binding-protein (OSBP)-related protein 4 binds 25-hydroxycholesterol and interacts with vimentin intermediate filaments. *Biochem. J.* 361 461–472. 10.1042/0264-6021:3610461 11802775 PMC1222328

[B141] Wang H. Sun H. Q. Zhu X. Zhang L. Albanesi J. Levine B. (2015). GABARAPs regulate PI4P-dependent autophagosome:lysosome fusion. *Proc. Natl. Acad. Sci. U.S.A.* 112 7015–7020. 10.1073/pnas.1507263112 26038556 PMC4460452

[B142] Wang Y. J. Wang J. Sun H. Q. Martinez M. Sun Y. X. Macia E. (2003). Phosphatidylinositol 4 phosphate regulates targeting of clathrin adaptor AP-1 complexes to the Golgi. *Cell* 114 299–310. 10.1016/s0092-8674(03)00603-2 12914695

[B143] Weber-Boyvat M. Kentala H. Lilja J. Vihervaara T. Hanninen R. Zhou Y. (2015a). OSBP-related protein 3 (ORP3) coupling with VAMP-associated protein A regulates R-Ras activity. *Exp. Cell Res.* 331 278–291. 10.1016/j.yexcr.2014.10.019 25447204

[B144] Weber-Boyvat M. Kentala H. Peränen J. Olkkonen V. M. (2015b). Ligand-dependent localization and function of ORP-VAP complexes at membrane contact sites. *Cell. Mol. Life Sci.* 72 1967–1987. 10.1007/s00018-014-1786-x 25420878 PMC11114005

[B145] Wijdeven R. H. Janssen H. Nahidiazar L. Janssen L. Jalink K. Berlin I. (2016). Cholesterol and ORP1L-mediated ER contact sites control autophagosome transport and fusion with the endocytic pathway. *Nat. Commun.* 7 11808–11814. 10.1038/ncomms11808 27283760 PMC4906411

[B146] Wilfling F. Thiam A. R. Olarte M.-J. Wang J. Beck R. Gould T. J. (2014). Arf1/COPI machinery acts directly on lipid droplets and enables their connection to the ER for protein targeting. *eLife* 3:e01607. 10.7554/eLife.01607 24497546 PMC3913038

[B147] Wong K. Meyers R. Cantley L. C. (1997). Subcellular locations of phosphatidylinositol 4-kinase isoforms. *J. Biol. Chem.* 272 13236–13241. 10.1074/jbc.272.20.13236 9148941

[B148] Wong L. H. Čopič A. Levine T. P. (2017). Advances on the transfer of lipids by lipid transfer proteins. *Trends Biochem. Sci.* 42 516–530. 10.1016/j.tibs.2017.05.001 28579073 PMC5486777

[B149] Wong L. H. Gatta A. T. Levine T. P. (2019). Lipid transfer proteins: the lipid commute via shuttles, bridges and tubes. *Nat. Rev. Mol. Cell Biol.* 20 85–101. 10.1038/s41580-018-0071-5 30337668

[B150] Wu H. Carvalho P. Voeltz G. K. (2018). Here, there, and everywhere: the importance of ER membrane contact sites. *Science* 361:eaan5835. 10.1126/science.aan5835 30072511 PMC6568312

[B151] Wyles J. P. Perry R. J. Ridgway N. D. (2007). Characterization of the sterol-binding domain of oxysterol-binding protein (OSBP)-related protein 4 reveals a novel role in vimentin organization. *Exp. Cell Res.* 313 1426–1437. 10.1016/j.yexcr.2007.01.018 17350617

[B152] Xu Y. Liu Y. Ridgway N. D. McMaster C. R. (2001). Novel members of the human oxysterol-binding protein family bind phospholipids and regulate vesicle transport. *J. Biol. Chem.* 276 18407–18414. 10.1074/jbc.M101204200 11279184

[B153] Zerial M. McBride H. (2001). Rab proteins as membrane organizers. *Nat. Rev. Mol. Cell Biol.* 2 107–117. 10.1038/35052055 11252952

[B154] Zewe J. P. Miller A. M. Sangappa S. Wills R. C. Goulden B. D. Hammond G. R. V. (2020). Probing the subcellular distribution of phosphatidylinositol reveals a surprising lack at the plasma membrane. *J. Cell Biol.* 219 253–219. 10.1083/jcb.201906127 32211893 PMC7054989

[B155] Zhao K. Ridgway N. D. (2017). Oxysterol-binding protein-related protein 1L regulates cholesterol egress from the endo-lysosomal system. *Cell Rep.* 19 1807–1818. 10.1016/j.celrep.2017.05.028 28564600

[B156] Zhao K. Foster J. Ridgway N. D. (2020). Oxysterol-binding protein-related protein 1 variants have opposing cholesterol transport activities from the endolysosomes. *Mol. Biol. Cell* 31 793–802. 10.1091/mbc.E19-12-0697 32023146 PMC7185962

[B157] Zhong W. Yi Q. Xu B. Li S. Wang T. Liu F. (2016). ORP4L is essential for T-cell acute lymphoblastic leukemia cell survival. *Nat. Commun.* 7 12702–12714. 27581363 10.1038/ncomms12702 PMC5025801

